# Risk of Rash in PD-1 or PD-L1-Related Cancer Clinical Trials: A Systematic Review and Meta-Analysis

**DOI:** 10.1155/2022/4976032

**Published:** 2022-07-18

**Authors:** Yuan Tian, Chi Zhang, Qi Dang, Kaiyong Wang, Qian Liu, Hongmei Liu, Heli Shang, Junyan Zhao, Yuedong Xu, Tong Wu, Wei Liu, Xiaowei Yang, Mohammed Safi

**Affiliations:** ^1^Radiotherapy Department, Shandong Second Provincial General Hospital, Jinan, Shandong 250023, China; ^2^Department of Cardiology, The Second Hospital, Cheeloo College of Medicine, Shandong University, Jinan, Shandong 250033, China; ^3^Phase I Clinical Trial Center, Shandong Cancer Hospital and Institute, Shandong First Medical University and Shandong Academy of Medical Sciences, Jinan, Shandong 250012, China; ^4^Department of Respiratory and Critical Care Medicine, The People's Hospital of Yuncheng County, Heze, Shandong 274799, China; ^5^Department of Oncology, Jinan Central Hospital, Weifang Medical University, Jinan, Shandong 250013, China; ^6^Radiotherapy Oncology Department, The First Affiliated Hospital of Shandong First Medical University and Shandong Provincial Qianfoshan Hospital, Shandong Key Laboratory of Rheumatic Disease and Translational Medicine, Shandong Lung Cancer Institute, Jinan 250014, Shandong, China; ^7^Nursing Department, The First Affiliated Hospital of Shandong First Medical University & Shandong Provincial Qianfoshan Hospital, Jinan, Shandong 250014, China; ^8^Endocrinology Department, Shandong Provincial Qianfoshan Hospital, The First Hospital Affiliated with Shandong First Medical University, Jinan 250014, Shandong, China; ^9^Department of Hepatobiliary Intervention, Beijing Tsinghua Changgung Hospital, School of Clinical Medicine, Tsinghua University, Beijing 102218, China; ^10^Department of Oncology, First Affiliated Hospital of Dalian Medical University, Dalian 116021, China

## Abstract

**Background:**

Given that immune-related rash was the most frequently reported PD-1 or PD-L1-related skin toxicity, this systematic review and meta-analysis were conducted to elucidate its incidence risk.

**Methods:**

The meta-analysis was carried out according to the PRISMA guidelines. The random effect model was used in the process of all analyses. Skin rash of all grades and grades 3–5 were calculated and gathered in the final comprehensive analyses.

**Results:**

The study included 86 clinical trials classified into 15 groups. Compared with chemotherapy, PD-1 or PD-L1 inhibitors significantly strengthened the risk of developing rash across all grades (OR = 1.66, 95% CI: [1.31, 2.11]; *p* < 0.0001). This trend was significantly stronger when the control group was placebo (OR = 2.62, 95% CI: [1.88, 3.65]; *p* < 0.00001). Similar results were observed when PD-1 or PD-L1 inhibitors were given together with chemotherapy (OR = 1.87, 95% CI: [1.59, 2.20]; *p* < 0.00001), even in patients with grades 3–5. As with other combination therapies, the risk of developing rash for all grades was enhanced when PD-1 or PD-L1 was given together with chemotherapy as the second-line option (OR = 2.98, 95% CI: [1.87, 4.75]; *p*=0.05). No statistically significant differences could be found in skin rash between the PD-1 and PD-L1-related subgroups.

**Conclusion:**

Whether PD-1 or PD-L1 inhibitors were given alone or together with others, the risk of developing rash would be enhanced. Furthermore, the risk of developing rash appeared to be higher when PD-1 or PD-L1 inhibitors together with other antitumor drugs were given as the second-line options. No statistically significant results of developing rash between PD-1 and PD-L1 subgroups were obtained owing to the participation of PD-1 or PD-L1 inhibitors.

## 1. Introduction

Due to tobacco cessation, advancements in early diagnosis and treatment, the death rate of various cancers has been falling year after year in the United States, while the survival rate has been improving, particularly for non-small-cell lung cancer (NSCLC) [1]. Among the several therapeutic options available, cancer immunotherapy is extremely successful in increasing cancer patients' survival rates, particularly when PD-1 or PD-L1 inhibitors are given [2]. On the basis of research into the mechanisms of immune escape, PD-1 or PD-L1 inhibitors have reshaped the therapy landscape for cancer by activating the immune system, while also gradually reporting plenty of treatment-related side effects [3]. Although the association between some adverse events and PD-1 or PD-L1 inhibitors has been extensively examined and documented [4–9], many toxicities remain unexplored, including skin toxicities [3].

Skin toxicities, such as rash, pruritus, vitiligo, palmar-plantar erythrodysasthesia (PPE), erythema, eczema, urticaria, dermatitis, dry skin, and maculopapular rash, were frequently observed in cancer patients treated with PD-1 or PD-L1 [3, 10, 11]. Additionally, autoimmune skin toxicities associated with PD-1 or PD-L1 have been reported to be significantly more prevalent in patients with NSCLC who are in complete or partial remission [10]. This pattern may also be observed in other types of tumors [11, 12]. Correlations between adverse events and clinical benefit are not uncommon [13–15]. However, the correlations between the risk of developing skin toxicities and PD-1 or PD-L1 inhibitors, as well as their effect on patient prognosis, remain unknown. Therefore, the rash with the highest rate of occurrence among PD-1 or PD-L1-related skin toxicities was chosen for the comprehensive analysis. To begin, subgroup analysis would be used to assess the difference in rash risk between the PD-1 and PD-L1 subgroups; second, the effect of different administration timing on rash would be assessed; and then, detailed subgroup analysis would be used to elucidate the source of heterogeneity.

## 2. Methods

The design and specific procedures of the meta-analysis were carried out step-by-step as recommended by the PRISMA [16].

### 2.1. Eligibility Screening for All Clinical Trials

Phase III clinical trials involving PD-1 or PD-L1 inhibitors with control groups would be preferred. Other clinical trials with control groups would be placed in an alternate location. With the exception of hematological malignancies, the types of solid tumors would not be limited. All data involving rash would be extracted and recorded in preparation for the subsequent adequate subgroup analysis. Four authors were appointed for eligibility screening.

### 2.2. Formulation and Implementation of Literature Search Strategy

According to the principle of PICOS (participants, interventions, comparisons, outcomes, and study design), the specific strategy of literature search was specified and implemented by all authors [16]. First, neoplasm was firstly searched as the MeSH keyword, not limited to specific solid tumor types. Then, all kinds of PD-1 or PD-L1 inhibitors, including common names, trade names, and abbreviations, would be searched as keywords and the search results would be unioned.

The publication time of relevant studies would be limited from July 09, 2013, to September 14, 2021. If one clinical trial was repeatedly reported several times, only the one with full detailed data could be selected for the analysis.

### 2.3. Quality Evaluation and Publication Bias Screening

The revised Cochrane Collaboration tool was adopted for bias risk screening in all selected trials [17], and the Funnel plot and Egger's test were used for publication bias assessments [18]. A *p* value < 0.05 was considered as the evidence for the existence of publication bias.

The quality screening of all the enrolled clinical trials were also carried out by the above four authors. The screening criteria were listed as the following 5 items: (a) selection bias, (b) performance bias, (c) detection bias, (d) attrition bias, and (e) reporting bias [17].

### 2.4. Screening of Results

The main outcome measure was the risk of PD-1 or PD-L1 involving rash across all grades, while the second was the rash for grades 3–5. The main information of all trials would be extracted and summarized in the single table (Table 1). The main content included in the table was listed as the following items: the first author's name, publication years, trial title, registered trial number, therapies lines, treatment regimens, participants, phase, tumor type, RCT, and the number of rash events.

### 2.5. Heterogeneity Screening and Statistical Analyses

Cochrane's Q and I^2^ statistics were used for heterogeneity screening, as described by Higgins and colleagues [16, 19], while the Harbord test was used for publication bias evaluation [19]. Three grades of heterogeneity were defined according to the I^2^ value: The two separation thresholds were 25% and 50%, respectively [20]. Using Review Manager 5.3, odds ratios (OR) and 95% confidence intervals (CI) across all enrolled clinical trials using the random effect (RE) method were calculated [21], whereas funnel plots were constructed using the fixed effect (FE) model. All statistical tests were two-sided, and *p* < 0.05 was taken as a statistically significant result. In the process of analyses, adequate subgroup evaluations would be carried out according to the actual situation.

## 3. Results

### 3.1. Literature Search Results

**Figure 1:**
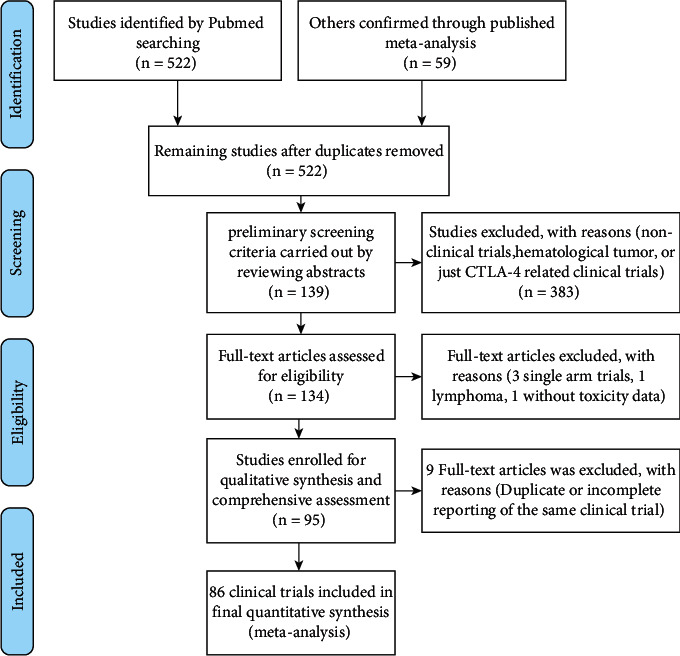
The flow diagram of all enrolled clinical trials.

After a preliminary PubMed search, 522 studies were retrieved (Figure 1). After criteria screened, 95 studies involving 86 clinical trials, including 55207 participants, were used for the final comprehensive analyses [22–25], [26–30], [31–35], [36–40], [41–45], [46–50], [51–55], [56–60], [61–65], [66–70], [71–75], [76–80], [81–85], [86–90], [91–95], [96–110], [111–115], [116, 117]. According to the PICOS guidelines, the detailed process of literature screening was provided in the form of PRISMA flow diagram (Figure 1). All types of literature included in the quality checking were finished by the four authors independently and finally summarized by the corresponding author and then plotted as the (S Figure 1) [22–25], [26–30], [31–35], [36–40], [41–45], [46–50], [51–55], [56–60], [61–65], [66–70], [71–75], [76–80], [81–85], [86–90], [91–95], [96–110], [111–115], [116, 117].

### 3.2. Basic Information for All Included Clinical Trials

Basic characteristics of 86 clinical trials included in the study were extracted and shown in Table 1 [5], [22–25], [26–30], [31–35], [36–40], [41–45], [46–50], [51–55], [56–60], [61–65], [66–70], [71–75], [76–80], [81–85], [86–90], [91–95], [96–110], [111–115], [116, 117]. 6 clinical trials, including KEYNOTE-021 [27, 28], KEYNOTE-189 [40–42], CheckMate 227 [47, 48], JAVELIN Renal 101 [51, 52], KEYNOTE-042 [61, 62], and CheckMate 067 [114–117], were repeatedly reported multiple times by different reporters, and only one with the detailed data could be selected for the final analyses. Among them, there were 72 Phase III, 8 Phase II, 2 Phase II/III, 1 Phase I/II, 1 Phase I/III, 1 Phase Ib, and 1 Phase I clinical trials. In 55 clinical trials, PD-1 or PD-L1 inhibitors were given alone or together with other antitumor drugs as the first-line regimens [23, 27, 28, 31], [36–38, 40–42], [44–48, 50, 53], [55–58], [61–68], [72, 73, 75], [77–80, 83–86], [88–95], [98–104], [106, 107, 109, 111, 112], [114–117], while previous therapies were found in the other 31 clinical trials [22, 24–26, 29, 30, 32–35, 39, 43, 49, 51, 52, 54, 59, 60, 69–71, 74, 76, 81, 82, 87, 96, 97, 105, 108, 110, 113]. Among the tumor types involved in all enrolled clinical trials, NSCLC accounted for the highest proportion (*n* = 22) [22, 24, 26–28, 35–37, 39–43, 47, 48, 53, 58, 59, 61, 62, 68, 74, 75, 92, 93, 98, 113], followed by melanoma (*n* = 11) [23, 31, 34, 44, 57, 65, 67, 76, 81, 104, 114–117], urothelial carcinoma (*n* = 8) [32, 49, 71, 77, 78, 86, 87, 90], renal cell carcinoma (*n* = 7) [25, 51, 52, 55, 56, 89, 91, 102], SCLC (*n* = 7) [29, 38, 50, 79, 95, 100, 105], triple-negative breast cancer (*n* = 6) [46, 66, 73, 82, 84, 88], and head and neck squamous cell carcinoma (*n* = 4) [30, 60, 63, 84].

All enrolled clinical trials were classified into 15 groups in view of the treatment regimens of all the control groups, which were listed as follows: Group A (PD-1 or PD-L1 versus Chemotherapy) [22–24, 26, 32, 43, 47, 49, 54, 59, 61, 68, 77, 78, 80, 82, 86, 92, 96], Group B (PD-1 or PD-L1 plus Chemotherapy versus Chemotherapy) [27, 37, 41, 45, 53, 66, 73, 74, 77, 79, 80, 84, 86, 95, 96, 100, 101, 103, 107, 111, 112], Group C (Camrelizumab plus Chemotherapy versus Chemotherapy) [75, 99, 106], Group D (PD-1 or PD-L1 plus Chemotherapy plus Bevacizumab versus Chemotherapy plus Bevacizumab) [36, 107], Group E (PD-1 or PD-L1 versus Placebo) [33, 39, 44, 71, 87, 90, 91, 97, 98, 105, 108], Group F (PD-1 or PD-L1 plus Chemotherapy versus PD-1 or PD-L1) [63, 77, 80, 96], Group G (PD-1 or PD-L1 plus CTLA-4 versus PD-1 or PD-L1) [29, 47, 76, 78, 105, 118], Group H (PD-1 or PD-L1 versus CTLA-4) [34, 67, 117], Group I (PD-1 or PD-L1 plus CTLA-4 versus Chemotherapy) [47, 94], Group J (PD-1 or PD-L1 plus CTLA-4 plus Chemotherapy versus Chemotherapy) [93, 95], Group K (PD-1 or PD-L1 plus Bevacizumab versus Sorafenib) [64, 85], Group L (PD-1 or PD-L1 plus CTLA-4 versus CTLA-4) [31, 117], Group M (PD-1 or PD-L1 versus Methotrexate/docetaxel/cetuximab) [30, 60], and Group N (PD-1 or PD-L1 plus Antineoplastic Drug versus Sunitinib) [51, 55, 56, 89, 91]. The others would just be used for the systematic review [25, 26, 34, 57, 63, 65, 69, 72, 81, 83, 88, 89, 95, 101, 104, 105, 109, 110]. Within each group, the differences between the PD-1 and PD-L1 subgroups would be assessed firstly, followed by the treatment lines.

### 3.3. Risk of Bias

**Figure 2:**
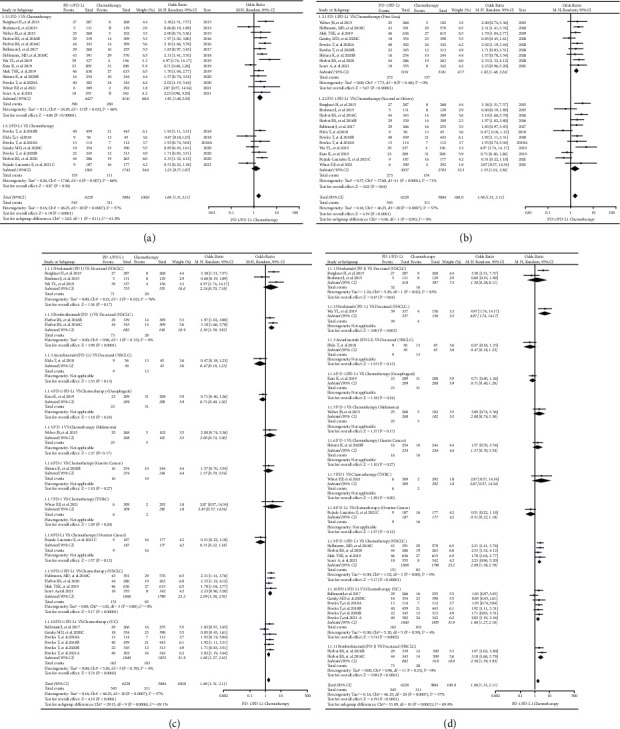
Forest plots of comparison in Group A (PD-1 or PD-L1 versus Chemotherapy). (a) The OR of rash for all grades calculated by the random effect (RE) model: subgroup analyses were performed according to the types of immune checkpoint inhibitors (PD-1 or PD-L1). (b) The OR of rash for all grades calculated by the random effect (RE) model: subgroup analyses were performed according to the treatment lines (first or second line). (c) The OR of rash for all grades calculated by the random effect (RE) model: subgroup analyses were performed based on drug name, tumor type, and immune checkpoint type. (d) The OR of rash for all grades calculated by the random effect (RE) model: subgroup analyses were performed based on drug name, tumor type, immune checkpoint type, and I2 value.

**Figure 3:**
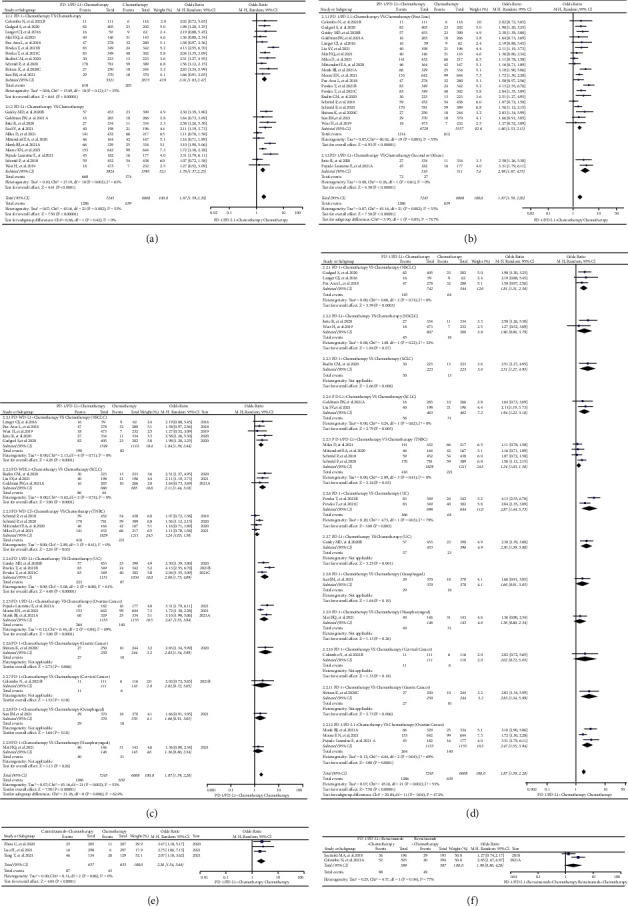
Forest plots of comparison in combination regimens. (a) The OR of rash for all grades checked using the random effect (RE) model in Group B (PD-1 or PD-L1 plus Chemotherapy versus Chemotherapy): subgroup analyses were carried out according to the types of immune checkpoint inhibitors (PD-1 or PD-L1). (b) The OR of rash for all grades checked using the random effect (RE) model in Group B (PD-1 or PD-L1 plus Chemotherapy versus Chemotherapy): subgroup analyses were carried out according to the treatment lines (first or second line). (c) The OR of rash for all grades checked using the random effect (RE) model in Group B (PD-1 or PD-L1 plus Chemotherapy versus Chemotherapy): subgroup analyses were carried out based on tumor type. (d) The OR of rash for all grades checked using the random effect (RE) model in Group B (PD-1 or PD-L1 plus Chemotherapy versus Chemotherapy): subgroup analyses were carried out based on tumor type and immune checkpoint type. (e) The OR of rash for all-grade checked using the random effect (RE) model in Group C (Camrelizumab plus Chemotherapy versus Chemotherapy). (f) The OR of rash for all-grade checked using the random effect (RE) model in Group D (PD-1 or PD-L1 plus Chemotherapy plus Bevacizumab versus Chemotherapy plus Bevacizumab).

**Figure 4:**
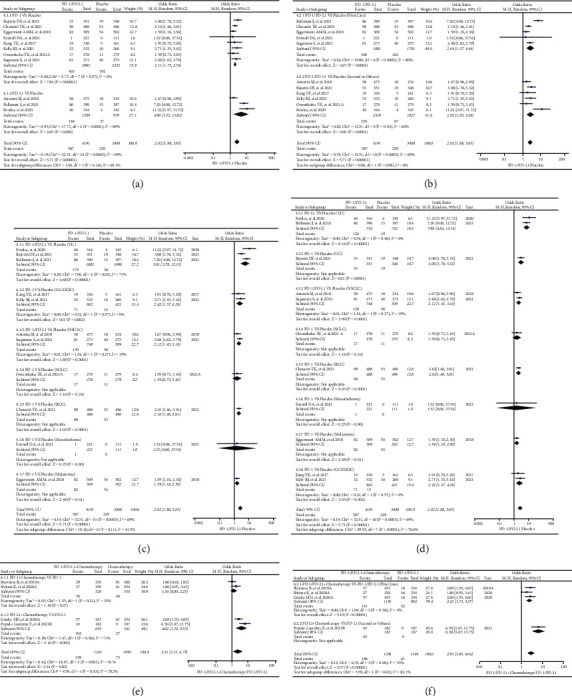
Forest plots of different comparison groups. (a) The OR of rash for all grades checked using the random effect (RE) model in Group E (PD-1 or PD-L1 versus Placebo): subgroup analyses were carried out according to the types of immune checkpoint inhibitors (PD-1 or PD-L1). (b) The OR of rash for all grades checked using the random effect (RE) model in Group E (PD-1 or PD-L1 versus Placebo): subgroup analyses were carried out according to the treatment lines (first or second line). (c) The OR of rash for all grades checked using the random effect (RE) model in Group E (PD-1 or PD-L1 versus Placebo): subgroup analyses were carried out based on tumor type. (d) The OR of rash for all grades checked using the random effect (RE) model in Group E (PD-1 or PD-L1 versus Placebo): subgroup analyses were carried out based on tumor type and I2 value. (e) The OR of rash for all grades checked using the random effect (RE) model in Group F (PD-1 or PD-L1 plus Chemotherapy VS PD-1 or PD-L1): subgroup analyses were carried out according to the types of immune checkpoint inhibitors (PD-1 or PD-L1). (f) The OR of rash for all grades checked using the random effect (RE) model in Group F (PD-1 or PD-L1 plus Chemotherapy versus PD-1 or PD-L1): subgroup analyses were carried out according to the treatment lines (first or second line).

**Figure 5:**
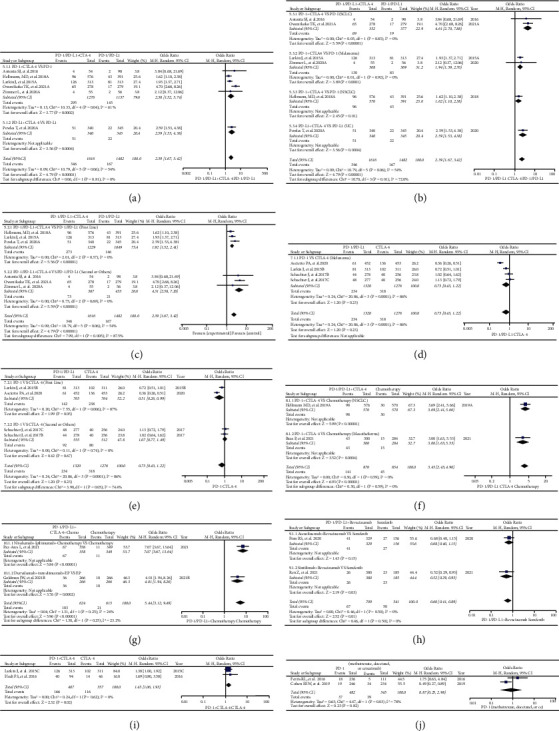
Forest plots of comparison groups (Groups G-M). (a) The OR of rash for all grades checked using the random effect (RE) model in Group G (PD-1 or PD-L1 plus CTLA-4 versus PD-1 or PD-L1): subgroup analyses were carried out according to the types of immune checkpoint inhibitors (PD-1 or PD-L1). (b) The OR of rash for all grades checked using the random effect (RE) model in Group G (PD-1 or PD-L1 plus CTLA-4 versus PD-1 or PD-L1): subgroup analyses were carried out based on tumor type. (c) The OR of rash for all grades checked using the random effect (RE) model in Group G (PD-1 or PD-L1 plus CTLA-4 versus PD-1 or PD-L1): subgroup analyses were carried out according to the treatment lines (first or second line). (d) The OR of rash for all grades checked using the random effect (RE) model in Group H (PD-1 or PD-L1 versus CTLA-4). (e) The odds ratio of rash for all grades calculated by the random effect (RE) model in Group H (PD-1 or PD-L1 versus CTLA-4): subgroup analyses were carried out according to the treatment lines (first or second line). (f) The OR of rash for all-grade checked using the random effect (RE) model in Group I (PD-1 or PD-L1 plus CTLA-4 versus Chemotherapy): subgroup analyses were carried out based on tumor type. (g) The OR of rash for all grades checked using the random effect (RE) model in Group J (PD-1 or PD-L1 plus CTLA-4 plus Chemotherapy versus Chemotherapy): subgroup analyses were carried out based on treatment regimens. (h) The OR of rash for all grades checked using the random effect (RE) model in Group K (PD-1 or PD-L1 plus Bevacizumab versus Sorafenib): subgroup analyses were carried out according to the types of immune checkpoint inhibitors (PD-1 or PD-L1). (i) The odds ratio of rash for all-grade checked using the random effect (RE) model in Group L (PD-1 or PD-L1 plus CTLA-4 versus CTLA-4). (j) The odds ratio of rash for all grades checked using the random effect (RE) model in Group M (PD-1 or PD-L1 versus Methotrexate/docetaxel/cetuximab).

**Figure 6:**
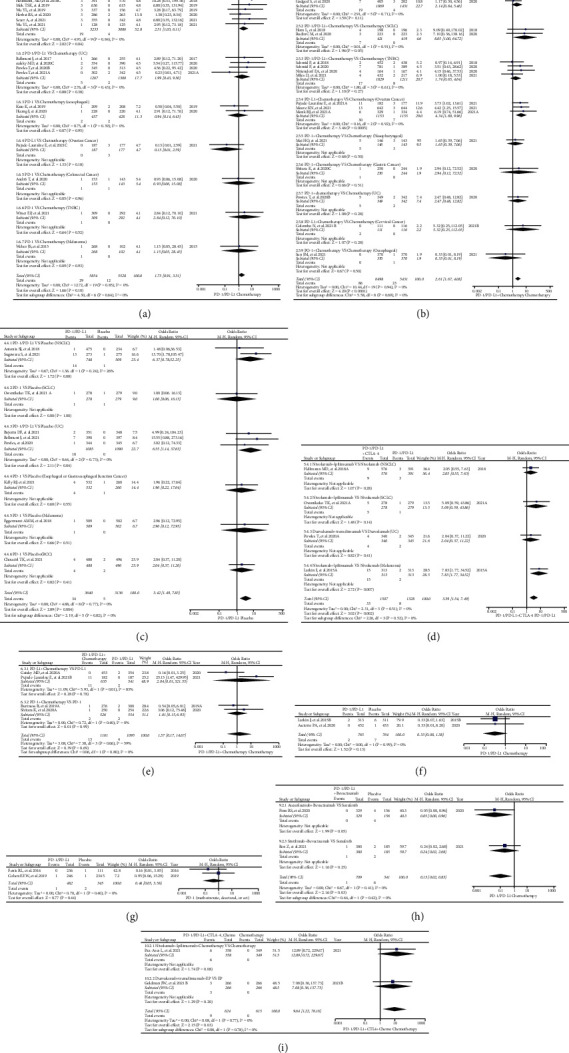
Forest plots of comparison groups for grades 3–5. (a) The OR of rash for grades 3–5 checked using the random effect (RE) model in Group A (PD-1 or PD-L1 versus Chemotherapy): subgroup analyses were carried out based on tumor types. (b) The OR of rash for grades 3–5 checked using the random effect (RE) model in Group B (PD-1 or PD-L1 plus Chemotherapy versus Chemotherapy): subgroup analyses were carried out based on tumor types. (c) The OR of rash for grades 3–5 checked using the random effect (RE) model in Group E (PD-1 or PD-L1 versus Placebo): subgroup analyses were carried out based on tumor types. (d) The OR of rash for grades 3–5 checked using the random effect (RE) model in Group G (PD-1 or PD-L1 plus CTLA-4 versus PD-1 or PD-L1): subgroup analyses were carried out based on tumor types. (e) The OR of rash for grades 3–5 checked using the random effect (RE) model in Group F (PD-1 or PD-L1 plus Chemotherapy versus PD-1 or PD-L1): subgroup analyses were carried out based on the types of immune checkpoint inhibitors (PD-1 or PD-L1). (f) The OR of rash for grades 3–5 checked using the random effect (RE) model in Group H (PD-1 or PD-L1 versus CTLA-4). (g) The odds ratio of rash for grades 3–5 checked using the random effect (RE) model in Group M (PD-1 or PD-L1 versus Methotrexate/docetaxel/cetuximab). (h) The OR of rash for grades 3–5 checked using the random effect (RE) model in Group K (PD-1 or PD-L1 plus Bevacizumab versus Sorafenib): subgroup analyses were carried out based on the name of immune checkpoint inhibitors. (i) The OR of rash for grades 3–5 checked using the random effect (RE) model in Group J (PD-1 or PD-L1 plus CTLA-4 plus Chemotherapy versus Chemotherapy): subgroup analyses were carried out based on treatment regimens.

86 clinical trials, involving 95 literatures, were all screened for 5 relevant bias risks, and the results were shown in the (S Figure 1) [22–25], [26–30], [31–35], [36–40], [41–45], [46–50], [51–55], [56–60], [61–65], [66–70], [71–75], [76–80], [81–85], [86–90], [91–95], [96–110], [111–115], [116, 117]. Data with high bias would not be adopted for the final meta-analysis (S Figure 1) [57, 114–116]. The funnel plots for publication bias assessments were constructed and shown in the corresponding figures (S Figures 2–6).

### 3.4. Risk Assessments of Rash for All Grades in Group A (PD-1 or PD-L1 versus Chemotherapy)

Reactive cutaneous capillary endothelial proliferation (RCCEP) was the characteristic rash of camrelizumab, so the clinical trials including camrelizumab were evaluated separately [70]. 19 clinical trials in Group A were summarized and prepared for the final analyses [22–24, 26, 32, 43, 47, 49, 54, 59, 61, 68, 77, 78, 80, 82, 86, 92, 96]. Among all tumor types, NSCLC was the most common one (*n* = 10) [22, 24, 26, 43, 47, 59, 61, 68, 92], followed by UC (*n* = 5) [32, 49, 77, 78, 86].

Through analyses, we found that PD-1 or PD-L1 inhibitors significantly increased the risk of developing rash for all grades (OR = 1.66, 95% CI: [1.31, 2.11]; I^2^ = 57%, *Z* = 4.19, *p* < 0.0001; Figures 2(a)–2(d)). Compared with the PD-L1 subgroup, the risk of developing rash appeared to be higher in PD-1 subgroup (OR = 1.92, 95% CI: [1.48, 2.50]; I^2^ = 46%, *Z* = 4.86, *p*=0.03; Figure 2(a)). Similar trend was also found when subgroup was divided based on the treatment lines (OR = 1.82, 95% CI: [1.48, 2.24]; I^2^ = 0%, *Z* = 5.67, *p* < 0.00001; Figure 2(b)). However, no statistically significant subgroup differences were found in the above two subgroups (Chi^2^ = 2.62, *p*=0.11, I^2^ = 61.8%, Figure 2(a); Chi^2^ = 0.46, *p*=0.50, I^2^ = 0%, Figure 2(b)).

High heterogeneity (I^2^ = 57%) could be found in the analysis results (Figures 2(a)–2(d)). After adequate subgroup analyses, it was found that this high degree of heterogeneity stemmed mainly from the two clinical trials of NSCLC (I^2^ = 76%, Figure 2(c); I^2^ = 83%, Figure 2(d)) [22, 24]. The funnel plots of them are shown in S Figures 2(a)–2(d).

### 3.5. Risk Assessments of Rash for All Grades in Group B, Group C, and Group D

21 clinical trials in Group B were enrolled for the final analysis [27, 37, 41, 45, 53, 66, 73, 74, 77, 79, 80, 84, 86, 95, 96, 100, 101, 103, 107, 111, 112]. Among all enrolled clinical trials, clinical trials involving NSCLC (*n* = 5) still accounted for the highest proportion [27, 37, 41, 53, 74], followed by triple-negative breast cancer (TNBC) (*n* = 4) [45, 66, 73, 84], small cell lung cancer (SCLC) (*n* = 3) [79, 95, 100], ovarian cancer (OC) (*n* = 3) [96, 101, 103], and urothelial carcinoma (UC) (*n* = 2) [77, 86].

Compared with chemotherapy in Group B, it was found that PD-1 or PD-L1 together with chemotherapy significantly increased the risk of rash for all grades (OR = 1.87, 95% CI: [1.59, 2.20]; I^2^ = 53%, *Z* = 7.50, *p* < 0.00001; Figures 3(a)–3(d)), even in each evaluable subgroups (Figures 3(c) and 3(d)). Similar to the former analysis result of Group A, the PD-1 subgroup appeared to have a higher risk of rash (OR = 2.01, 95% CI: [1.63, 2.47]; Figure 3(a)) with no statistical significant differences [27, 37, 41, 73, 79, 80, 86, 107, 111, 112], when it was compared to the PD-L1 subgroup (Chi^2^ = 0.66, *p*=0.42; Figure 3(a)) [45, 53, 66, 74, 77, 84, 95, 96, 100, 101, 103]. Different from the previous analyses (Figure 2(b)), the incidence risk of rash was higher when PD-1 or PD-L1 together with chemotherapy was given as the second-line option (OR = 2.98, 95% CI: [1.87, 4.75]; Chi^2^ = 3.95, *p*=0.05; Figure 3(b)) [74, 96]. Subgroup analyses indicated that the incidence risk of rash was different among different tumor types, especially in UC subgroup (OR = 2.66, 95% CI: [1.73, 4.09]; I^2^ = 61%, *Z* = 4.48, *p* < 0.00001; Figure 3(c)) [77, 86]. Through subgroup analyses (Figures 3(c) and 3(d)), it was found that the high heterogeneity (I^2^ = 53%) might be mainly derived from the clinical trial KEYNOTE-361 (Figure 3(d)) [86].

Similar to the analysis result in Group B, the incidence risk of rash was also significantly increased when camrelizumab was given together with chemotherapy (OR = 2.30, 95% CI: [1.54, 3.44]; I^2^ = 0%, *Z* = 4.04, *p* < 0.0001; Figure 3(e)) [75, 99, 106]. However, when PD-1 or PD-L1 was given with bevacizumab and chemotherapy, no statistically significant analysis result was found (OR = 1.90, 95% CI: [0.86, 4.20]; I^2^ = 77%, *Z* = 1.60, *p*=0.11; Figure 3(e)). All the corresponding funnel lots are shown in S Figures 3(a)–3(f).

### 3.6. Risk Assessments of Rash for All Grades in Groups E and F

11 clinical trials in Group E were enrolled for the final analyses [33, 39, 44, 71, 87, 90, 91, 97, 98, 105, 108]. Among all clinical trials, clinical trials involving UC (*n* = 3) accounted for the highest proportion [71, 87, 90], followed by NSCLC (*n* = 2) [39, 98]. In 5 clinical studies [44, 90, 91, 98, 108], PD-1 or PD-L1 inhibitors were given as the first-line choice, whereas they were utilized as second-line or alternative therapeutic choices in the other 6 trials [33, 39, 71, 87, 97, 105].

Compared with placebo, it was found that PD-1 or PD-L1 inhibitors significantly increased the risk of developing rash for all grades (OR = 2.62, 95% CI: [1.88, 3.65]; I^2^ = 69%, *Z* = 5.71, *p* < 0.00001; Figures 4(a)–4(d)), especially for UC (OR = 5.81, 95% CI: [2.78, 12.15]; I^2^ = 71%, *Z* = 4.68, *p* < 0.00001; Figure 4(d)) [71, 87, 90]. Subgroup comparison indicated that the risk of developing rash was higher in the PD-L1 subgroup and first-line subgroup (Figures 4(a)–4(d)), which no statistical subgroup difference could be found. Overall heterogeneity in high degree (I^2^ = 69%) could be found, which was mainly caused by the clinical trial CheckMate 274 (I^2^ = 0%, Figure 4(c); I^2^ = 71%, Figure 4(d)) [87]. The corresponding funnel plots are shown in S Figures 4(a)–4(d).

4 clinical trials in Group F were enrolled for the final analyses [63, 77, 80, 96]. For PD-1/PD-L1 alone, the risk of rash was significantly increased when they were given with chemotherapy (OR = 2.33, 95% CI: [1.15, 4.75]; I^2^ = 81%, *Z* = 2.34, *p*=0.02; Figures 4(e) and 4(f)). Furthermore, this trend was much more pronounced when PD-L1 was combined with chemotherapy (OR = 4.02, 95% CI: [1.70, 9.53]; I^2^ = 71%, *Z* = 3.16, *p*=0.002; Figure 4(e)) or prescribed as the second line (OR = 6.50, 95% CI: [3.07, 13.75]; Figure 4(f)). Through subgroup analysis, it could be indicated that the high degree heterogeneity might be caused by the clinical trial JAVELIN Ovarian 200 (Figures 4(e) and 4(f)) [96]. The corresponding funnel plots were constructed and are shown in S Figures 4(e) and 4(f).

### 3.7. The Incidence Risk of Rash for All Grades in Groups G–N

6 clinical trials in Group G were used for the final analysis [29, 47, 76, 78, 105, 118]. In 3 clinical trials [47, 78, 118], PD-1 or PD-L1 inhibitors were given as the first-line choice, while they were used as second-line or other treatment options in the other 3 trials [29, 76, 105]. Compared with the adoption of PD-1 or PD-L1 inhibitor alone, the combination regimen (PD-1 or PD-L1 plus CTLA-4) significantly increased the risk of developing rash (OR = 2.39, 95% CI: [1.67, 3.42]; I^2^ = 54%, *Z* = 4.79, *p* < 0.00001; Figures 5(a)–5(c)). Subgroup analysis suggested that the risk of rash in SCLC was higher than that in other tumor types (OR = 4.61, 95% CI: [2.70, 7.88]; I^2^ = 0%, *Z* = 5.59, *p* < 0.00001; Figure 5(b)). Furthermore, the incidence risk of rash was higher when PD-1 or PD-L1 together with CTLA-4 was given as the second-line choice (OR = 4.31, 95% CI: [2.58, 7.20]; I^2^ = 0%, *Z* = 5.59, *p* < 0.00001; Figure 5(c)). By comprehensively evaluating the results of various subgroup analyses (Figures 5(a)–5(c)), we inferred that the high degree of heterogeneity might be mainly caused by the clinical trial CheckMate 227 [47]. The corresponding funnel plots are shown in S Figures 5(a)–5(c).

3 clinical trials in Group H (PD-1 or PD-L1 versus CTLA-4) were selected for the final meta-analysis [34, 67, 117]. The risk of developing rash caused by PD-1 was found to be significantly lower than that of CTLA-4 only in the first-line therapy subgroup (OR = 0.51, 95% CI: [0.26, 0.99]; I^2^ = 87%, *Z* = 1.99, *p*=0.05; Figure 5(e)), whereas the overall effect was not statistically significant (OR = 0.73, 95% CI: [0.43, 1.22]; I^2^ = 86%, *Z* = 1.20, *p*=0.23; Figure 5(d)). The subgroup analysis suggested that the high heterogeneity might be mainly caused by CheckMate 238 and CheckMate 067 [67, 117]. The corresponding funnel plots are shown in S Figures 5(d) and 5(e).

For chemotherapy alone, PD-1 or PD-L1 together with CTLA-4 (Group I) [47, 94], or together with chemotherapy on this basis (Group J) [93, 95], would significantly increase the risk of developing rash (Figures 5(f) and 5(g)). However, the conclusion was still controversial due to few studies included in those analyses (Figures 5(f) and 5(g)). The corresponding funnel plots are shown in (S Figures 5(f) and 5(g)).

For sorafenib (Group K), the risk of developing rash was lower (OR = 0.60, 95% CI: [0.41, 0.89]; I^2^ = 0%, *Z* = 2.52, *p*=0.01; Figure 5(h)). When PD-1 or PD-L1 was given with CTLA-4 (Group L), the risk of developing rash was higher than that of CTLA-4 subgroup (OR = 1.43, 95% CI: [1.06, 1.93]; I^2^ = 0%, *Z* = 2.32, *p*=0.02; Figure 5(i)). When PD-1 was compared with chemotherapy (Group M), no statistical significant result was found (OR = 0.87, 95% CI: [0.25, 2.98]; I^2^ = 78%, *Z* = 0.23, *p*=0.82; Figure 5(j)). The corresponding funnel plots are shown in S Figures 5(k)–5(m).

In 5 of the 6 clinical trials of renal cell carcinoma, the control group was sunitinib [51, 55, 56, 89, 91]. In these 5 clinical trials, we found that PD-1 or PD-L1 increased the incidence risk of rash regardless of which antitumor drug was used in combination [51, 55, 56, 89, 91]. However, the meta-analysis could not be performed due to the lack of consistency in the experimental groups in these 5 clinical trials [51, 55, 56, 89, 91]. The types of combination therapy regimens involving PD-1 or PD-L1 have been increasingly used in different tumors [26, 34, 57, 63, 65, 69, 72, 81, 83, 88, 95, 101, 104, 105, 109, 110]. In those combined treatment regimens, rash has been reported, which further verified the correlation between PD-1 or PD-L1 and the incidence of rash [26, 34, 57, 63, 65, 69, 72, 81, 83, 88, 95, 101, 104, 105, 109, 110].

### 3.8. Risk Assessments of Rash for Grades 3–5

The risk of developing rash for grades 3–5 was reported in 18 clinical trials (Group A) [22–24, 26, 32, 47, 54, 59, 61, 68, 70, 72, 77, 78, 82, 86, 92, 96]. Through analyses, statistically significant result was found only in NSCLC (OR = 2.51, 95% CI: [1.03, 6.11]; I^2^ = 0%, *Z* = 2.02, *p*=0.04; Figure 6(a)) [22, 24, 47, 59, 61, 64, 68, 92], while the overall effect across all tumor types was not statistically different (OR = 1.73, 95% CI: [0.91, 3.31]; I^2^ = 0%, *Z* = 1.66, *p*=0.10; Figure 6(a)).

Similar to the risk of rash for all grades in Group B, the risk of developing rash was significantly higher than that of the control chemotherapy group [27, 36, 38, 41, 45, 53, 66, 73–75, 79, 80, 84, 86, 96, 101, 103, 107, 111, 112], when PD-1 or PD-L1 was given together with chemotherapy (OR = 2.61, 95% CI: [1.67, 4.08]; I^2^ = 0%, *Z* = 4.20, *p* < 0.0001; Figure 6(b)), especially for ovarian cancer (OR = 4.34, 95% CI: [1.89, 9.96]; I^2^ = 0%, *Z* = 3.46, *p*=0.0005; Figure 6(b)) [96, 101, 103]. The positive result could also be found in Group C (OR = 3.42, 95% CI: [1.49, 7.85]; I^2^ = 0%, *Z* = 2.89, *p*=0.004; Figure 6(c)), Group G (OR = 3.39, 95% CI: [1.54, 7.49]; I^2^ = 0%, *Z* = 3.02, *p*=0.002; Figure 6(d)), and Group J (OR = 9.64, 95% CI: [1.22, 76.16]; I^2^ = 0%, *Z* = 2.15, *p*=0.03; Figure 6(i)) [39, 44, 47, 77, 78, 81, 90, 91, 93, 95, 98, 105, 117]. However, when PD-1 or PD-L1 plus bevacizumab were compared with sorafenib, the risk of developing rash was lower than that of the control group (OR = 0.13, 95% CI: [0.02, 0.83]; I^2^ = 0%, *Z* = 2.16, *p*=0.03; Figure 6(h)). In the other groups, no statistical significant results could be found (Figures 6(e)–6(g)). All the corresponding funnel plots were constructed and are shown in S Figures 6(a)–6(i).

## 4. Discussion

Among several therapeutic options available, cancer immunotherapy is extremely successful in increasing tumor patients' survival rates, particularly with PD-1/PD-L1 inhibitors [2]. Currently, PD-1 or PD-L1 inhibitors are extensively employed in the treatment of many types of malignancies, and the combination regimens using PD-1 or PD-L1 inhibitors are diversified [22–25], [26–30], [31–35], [36–40], [41–45], [46–50], [51–55], [56–60], [61–65], [66–70], [71–75], [76–80], [81–85], [86–90], [91–95], [96–110], [111–115], [116, 117], [118]. As with cetuximab [119, 120], rash associated with therapeutic benefit was one of the most frequently reported skin toxicities associated with PD-1 or PD-L1 inhibitors [13–15]. The correlation between rash and PD-1 or PD-L1 inhibitors, on the other hand, has to be further clarified in detail, particularly in diverse combination treatment regimens. Therefore, a systematic review and meta-analysis were conducted with the guidelines of the PRISMA criteria (Figure 1) [16].

After quality screening (S Figure 1), 86 clinical trials with complete data were adopted for the final comprehensive analyses [22–25], [26–30], [31–35], [36–40], [41–45], [46–50], [51–55], [56–60], [61–65], [66–70], [71–75], [76–80], [81–85], [86–90], [91–95], [96–110], [111–115], [116, 117], which avoided the high risk of attrition bias. With the development of clinical research, PD-1 or PD-L1 inhibitors have been increasingly prescribed as the first-line antitumor options (*n* = 51) [23, 27, 28], [31, 36–38], [40–42], [44–48], [50, 53], [55–58], [61–68], [72, 73], [75, 77–80], [83–86], [88–95], [98–104], [106, 107, 109], [111, 112], [114–117], especially for PD-1 or PD-L1 combined regimens [27, 36, 38, 41, 45, 53, 66, 73, 75, 79, 80, 84, 86, 99, 101, 103, 107, 111, 112], which also increase the difficulty of elucidating the relationship between PD-1 or PD-L1 and the risk of rash. Therefore, it is necessary for us to conduct this meta-analysis.

According to the compositions of all the control groups, all the enrolled clinical trials were firstly classified into different groups (Groups A-N), and then, analyses were carried out for each group (Figures 2–6 and S Figures 2–6). Through the analyses, it was found that PD-1 or PD-L1 inhibitors raised the risk of developing rash (Figure 2, Figures 4(a)–4(d), and Figure 6(a)), whether compared with chemotherapy or placebo alone (Group A and Group E) [22–24, 26, 32, 33, 39, 43, 44, 47, 49, 54, 59, 61, 68, 71, 77, 78, 80, 82, 86, 87, 90–92, 96–98, 105, 108]. However, this effect was weaker than CTLA-4 with no statistical significance (Group H) (Figures 5(d)–5(e) and 6(f)) [34, 67, 117]. In the combined antitumor treatment regimens containing PD-1 or PD-L1 inhibitors (Group B, Group C, Group D, and Group L) [27, 31, 36, 37, 41, 45, 53, 66, 73–75, 77, 79, 80, 84, 86, 95, 96, 99–101, 103, 106, 107, 111, 112, 117], it was also found that the risk of rash was increased due to the involvement of PD-1 or PD-L1 inhibitors (Figure 3, Figures 5(a)–5(c), Figure 6(b), S Figure 3, S Figure 5(a)–5(c), and S Figure 6(b)). Similar trend was also found in other PD-1 or PD-L1 inhibitor-based combination regimens (Group F, Group G, Group I, and Group G) (Figures 4(e) and 4(f); Figures 5(a)–5(c), 5(f), 5(g), 6(d), 6(e), and 6(i); S Figures 4(e)–4(f); S Figures 5(a)–5(c), 5(f), 5(g), 6(d), 6(e), and 6(i)) [29, 47, 63, 76–78, 80, 93–96, 105, 117]. In the other clinical trials for which meta-analysis could not be performed, the experimental group of PD-1 or PD-L1 inhibitors involved also indicated an increased risk of rash [25, 26, 34, 51, 55–57, 63, 65, 69, 72, 81, 83, 88, 89, 91, 95, 101, 104, 105, 109, 110]. From the above, it could be concluded that the risk of rash would be increased when PD-1 or PD-L-1 inhibitors were given alone or together with other antitumor regimens.

For the lack of head-to-head contrast between PD-1 and PD-L1 [22–25], [26–30], [31–35], [36–40], [41–45], [46–50], [51–55], [56–60], [61–65], [66–70], [71–75], [76–80], [81–85], [86–90], [91–95], [96–110], [111–115], [116, 117], we tried to investigate the differences between PD-1 and PD-L1 subgroups and indirectly observe the differences of rash risk. Although the analyses indicated that the risk of rash differed between PD-1 and PD-L1-related subgroups (Figures 2(a), 3(a) and 4(a)), no statistically significant results were found due to the involvement of PD-1 or PD-L1 inhibitors [22–24], [26, 27, 32], [33, 37, 39], [41, 43–45], [47, 49, 53], [54, 59, 61, 66], [68, 71–74], [77–80], [82, 84, 86], [87, 90–92], [95–98], [100, 101, 103], [105, 107, 108], [111, 112]. However, compared with the PD-1 involved subgroup (Figure 4(e)), the participation of chemotherapy significantly increased the risk of rash in the PD-L1 subgroup (*p*=0.03) [63, 77, 80, 96].

The similar strategy was used to elucidate the influence of PD-1 or PD-L1 involved treatment lines on the risk of developing rash (Figures 2(b), 3(b), 4(b), 4(f), 5(c), and 5(e)). Subgroup studies revealed an increased risk of rash when PD-1 or PD-L1 inhibitors were given together with other antitumor agents as the second-line choice (Figure 3(b), 4(f), and 5(c)) [27, 29, 37, 41, 45, 47, 53, 63, 66, 73, 74, 76–80, 84, 86, 95, 96, 100, 101, 103, 105, 107, 111, 112, 117]. When PD-1 or PD-L1 inhibitors were given alone, this incidence trend was only seen in Group H (Figure 5(e)) [34, 67, 117]. The reasons leading to the above results might be related to the combined treatment drugs, and the specific reasons were still need to be further studied.

The formation of heterogeneity is inevitable in the course of detailed examination (Figures 2–6). By conducting adequate subgroup analyses and comparing the results of rash between all grades and grades 3–5, the clinical trials responsible for the heterogeneity were identified, and further analyses revealed that the heterogeneity might be primarily due to the data themselves (Figure 6), implying that it would have little effect on the overall analysis results. Additionally, no noticeable publication bias was detected using funnel plots (S Figures 2–6). This further increased the reliability and rigor of this meta-analysis.

Although the correlation between skin toxicities and tumor regression had been reported frequently in some studies [10–12], no such data were found in all the enrolled clinical trials [22–25], [26–30], [31–35], [36–40], [41–45], [46–50], [51–55], [56–60], [61–65], [66–70], [71–75], [76–80], [81–85], [86–90], [91–95], [96–110], [111–115], [116, 117]. Therefore, to elucidate the correlation between the rash risk and tumor prognosis, more and more relevant clinical trials should be put into practice [13–15]. Furthermore, researchers needed to pay more attention to this kind of data and report it in a timely manner. In clinical work, we need to use treatment-related rashes cautiously to judge the treatment response and prognosis of patients.

## 5. Conclusions

The risk of developing rash would be enhanced whether PD-1 or PD-L1 inhibitors were given alone or together with others. Furthermore, the incidence risk of rash appeared to be higher when PD-1 or PD-L1 inhibitors together with other antitumor drugs were given as the second-line choice. No statistically significant differences in the results of the rash between the PD-1 and PD-L1 subgroups were found due to the involvement of PD-1 or PD-L1 inhibitors.

## Figures and Tables

**Table 1:** Basic information of all selected clinical trials.

Trial no.	Reference	NCT number	Drug	Treatment Regimens	Involving Patients	Rash	Previous therapy	Phase	Tumor Type
1	Borghaei H, *et al.* 2015 [22]	NCT01673867 (CheckMate 057)	Nivolumab (PD-1)	Nivolumab versus Docetaxel	555	35	Yes	III	Advanced non-squamous NSCLC
2	Weber JS, *et al.* 2015 [23]	NCT01721746 (CheckMate 037)	Nivolumab (PD-1)	Nivolumab versus Dacarbazine/Paclitaxel plus Carboplatin	370	30	No	III	Advanced melanoma
3	Brahmer J, *et al.* 2015 [24]	NCT01642004 (CheckMate 017)	Nivolumab (PD-1)	Nivolumab versus Docetaxel	260	13	Yes	III	Advanced squamous cell NSCLC
4	Motzer RJ, *et al.* 2015 [25]	NCT01668784 (CheckMate 025)	Nivolumab (PD-1)	Nivolumab versus Everolimus	803	120	Yes	III	Advanced RCC
5	Herbst RS, *et al.* 2016A [26]	NCT01905657 (KEYNOTE-010)	Pembrolizumab (PD-1)	Pembrolizumab 2 mg/kg versus Pembrolizumab 10 mg/kg	991	73	Yes	II/III	Advanced NSCLC
	Herbst RS, *et al.* 2016B [26]			Pembrolizumab 2 mg/kg versus Docetaxel		43			
	Herbst RS, *et al.* 2016C [26]			Pembrolizumab 10 mg/kg versus Docetaxel		58			
6	Langer CJ, *et al.* 2016 [27]	NCT02039674 (KEYNOTE-021)	Pembrolizumab (PD-1)	Pembrolizumab plus Carboplatin plus Pemetrexed versus Carboplatin plus Pemetrexed	121	25	No	II	Advanced nonsquamous NSCLC
	Awad MM, *et al.* 2021 [28]								
7	Antonia SJ, *et al.* 2016 [29]	NCT01928394 (CheckMate 032)	Nivolumab (PD-1)	Nivolumab versus Nivolumab plus Ipilimumab	152	6	Yes	I/II	Recurrent SCLC
8	Ferris RL, *et al.* 2016 [30]	NCT02105636 (CheckMate 141)	Nivolumab (PD-1)	Nivolumab versus (Methotrexate, Docetaxel, or Cetuximab)	347	23	Yes	III	Recurrent HNSCC
9	Hodi FS, *et al.* 2016 [31]	NCT01927419 (CheckMate 069)	Nivolumab (PD-1)	Nivolumab plus Ipilimumab versus Ipilimumab	140	54	No	II	Advanced melanoma
10	Bellmunt J, *et al.* 2017 [32]	NCT02256436 (KEYNOTE-045)	Pembrolizumab (PD-1)	Pembrolizumab versus Chemotherapy	531	45	Yes	III	Advanced UC
11	Kang YK, *et al.* 2017 [33]	NCT02267343 (ONO-4538-12, ATTRACTION-2)	Nivolumab (PD-1)	Nivolumab versus Placebo	491	24	Yes	III	Advanced gastric or GJC
12	Schachter J, *et al.* 2017A [34]	NCT01866319 (KEYNOTE-006)	Pembrolizumab (PD-1)	Pembrolizumab every 2 weeks versus Pembrolizumab every 3 weeks	811	92	Yes	III	Advanced melanoma
	Schachter J, *et al.* 2017B [34]			Pembrolizumab every 2 weeks versus Ipilimumab		84			
	Schachter J, *et al.* 2017C [34]			Pembrolizumab every 3 weeks versus Ipilimumab		88			
13	Antonia SJ, *et al.* 2017 [35]	NCT02125461 (PACIFIC)	Durvalumab (PD-L1)	Durvalumab versus Placebo	709	50	Yes	III	Advanced, unresectable, stage III NSCLC
14	Socinski MA, *et al.* 2018 [36]	NCT02366143 (IMpower150)	Atezolizumab (PD-L1)	Atezolizumab plus Bevacizumab plus Carboplatin plus Paclitaxel (ABCP) versus Bevacizumab plus Carboplatin plus Paclitaxel (BCP)	787	72	No	III	Metastatic nonsquamous NSCLC
15	Paz-Ares L, *et al.* 2018 [37]	NCT02775435 (KEYNOTE-407)	Pembrolizumab (PD-1)	Pembrolizumab plus chemotherapy versus chemotherapy	558	79	No	III	Squamous NSCLC
16	Horn L, *et al.* 2018 [38]	NCT02763579 (IMpower133)	Atezolizumab (PD-L1)	Atezolizumab plus Carboplatin plus Etoposide versus Carboplatin plus Etoposide	394	57	No	III	Extensive-stage SCLC
17	Antonia SJ, *et al.* 2018 [39]	NCT02125461 (PACIFIC)	Durvalumab (PD-L1)	Durvalumab versus Placebo	709	76	Yes	III	Stage III NSCLC
18	Gandhi L, *et al.* 2018 [40]	NCT02578680 (KEYNOTE-189)	Pembrolizumab (PD-1)	Pembrolizumab plus Pemetrexed plus A platinum-based drug versus Pemetrexed plus A platinum-based drug	607	105	No	II	Metastatic nonsquamous NSCLC
	Gadgeel S, *et al.* 2020 [41]								
	Rodríguez-Abreu D, *et al.* 2021 [42]								
19	Hida T, *et al.* 2018 [43]	NCT02008227 (OAK)	Atezolizumab (PD-L1)	Atezolizumab versus Docetaxel	101	22	Yes	III	Advanced/metastatic NSCLC
20	Eggermont AMM, *et al.* 2018 [44]	NCT02362594	Pembrolizumab (PD-1)	Pembrolizumab versus Placebo	1011	136	No	III	Resected stage III melanoma
21	Schmid P, *et al.* 2018 [45]	NCT02425891 (IMpassion130)	Atezolizumab (PD-L1)	Atezolizumab plus Nab-paclitaxel versus Nab-paclitaxel	890	113	No	III	Unresectable locally advanced or metastatic TNBC
	Emens LA, *et al.* 2021 [46]								
22	Hellmann MD, *et al.* 2018A [47]	NCT02477826 (CheckMate 227)	Nivolumab (PD-1)	Nivolumab plus Ipilimumab versus Nivolumab	1537	139	No	III	Stage IV or recurrent NSCLC
	Hellmann MD, *et al.* 2018B [47]			Nivolumab plus Ipilimumab versus Chemotherapy (platinum doublet)		125			
	Hellmann MD, *et al.* 2018C [47]			Nivolumab versus Chemotherapy (platinum doublet)		72			
	Reck M, *et al.* 2021A [48]			Nivolumab plus Ipilimumab versus Nivolumab		139			
	Reck M, *et al.* 2021B [48]			Nivolumab plus Ipilimumab versus Chemotherapy (platinum doublet)		125			
	Reck M, *et al.* 2021C [48]			Nivolumab versus Chemotherapy (platinum doublet)		72			
23	Powles T, *et al.* 2018A [49]	NCT02302807 (IMvigor211)	Atezolizumab (PD-L1)	Atezolizumab versus Chemotherapy (vinflunine paclitaxel or docetaxel)	1128	20	YSE	III	Locally advanced or metastatic UC
	Powles T, *et al.* 2018B [49]			Atezolizumab versus Chemotherapy (vinflunine paclitaxel or docetaxel)		61			
24	Paz-Ares L, *et al.* 2019 [50]	NCT03043872 (CASPIAN)	Durvalumab (PD-L1)	Durvalumab plus EP versus EP	531	6	No	III	Extensive-stage SCLC
25	Motzer RJ, *et al.* 2019 [51]	NCT02684006 (JAVELIN Renal 101)	Avelumab (PD-L1)	Avelumab plus Axitinib versus Sunitinib	873	96	Yes	III	Advanced RCC
	Motzer RJ, *et al.* 2020 [52]								
26	West H, *et al.* 2019 [53]	NCT02367781 (IMpower130)	Atezolizumab (PD-L1)	Atezolizumab plus Carboplatin plus Nab-paclitaxel versus Carboplatin plus Nab-paclitaxel	705	25	No	III	Metastatic nonsquamous NSCLC
27	Kato K, *et al.* 2019 [54]	NCT02569242 (ATTRACTION-3)	Nivolumab (PD-1)	Nivolumab versus Paclitaxel/Docetaxel	417	54	Yes	III	Advanced OSCC
28	Motzer R, *et al.* 2019 [55]	NCT02231749 (CheckMate 214)	Nivolumab (PD-1)	Nivolumab plus Ipilimumab versus Sunitinib	1082	193	No	III	Advanced RCC
29	Rini BI, *et al.* 2019 [56]	NCT02420821 (IMmotion151)	Atezolizumab (PD-L1)	Atezolizumab plus Bevacizumab versus Sunitinib	907	128	No	III	Metastatic RCC
30	Sullivan RJ, *et al.* 2019 [57]	NCT01656642	Atezolizumab (PD-L1)	Atezolizumab plus Vemurafenib versus Atezolizumab plus Cobimetinib plus Vemurafenib	56	20	No	Ib	BRAF-mutated melanoma
31	Hellmann MD, *et al.* 2019A [58]	NCT02477826 (CheckMate 227)	Nivolumab (PD-1)	Nivolumab plus Ipilimumab versus Nivolumab	1537	139	No	III	Advanced NSCLC
	Hellmann MD, *et al.* 2019B [58]			Nivolumab plus Ipilimumab versus Chemotherapy (platinum doublet)		125			
	Hellmann MD, *et al.* 2019C [58]			Nivolumab versus Chemotherapy (platinum doublet)		72			
32	Wu YL, *et al.* 2019 [59]	NCT02613507 (CheckMate 078)	Nivolumab (PD-1)	Nivolumab versus Docetaxel	493	43	Yes	III	Advanced NSCLC
33	Cohen EEW, *et al.* 2019 [60]	NCT02252042 (KEYNOTE-040)	Pembrolizumab (PD-1)	Pembrolizumab versus (Methotrexate, Docetaxel, or Cetuximab)	480	53	Yes	III	Recurrent or metastatic HNSCC
34	Mok TSK, *et al.* 2019 [61]	NCT02220894 (KEYNOTE-042)	Pembrolizumab (PD-1)	Pembrolizumab versus Chemotherapy	1251	73	No	III	Locally advanced or metastatic NSCLC
	Wu YL, *et al.* 2021 [62]								
35	Burtness B, *et al.* 2019A [63]	NCT02358031 (KEYNOTE-048)	Pembrolizumab (PD-1)	Pembrolizumab versus Pembrolizumab plus Chemotherapy	863	59	No	III	Recurrent or Metastatic HNSCC
	Burtness B, *et al.* 2019B [63]			Pembrolizumab versus Cetuximab plus Chemotherapy		141			
	Burtness B, *et al.* 2019C [63]			Pembrolizumab plus Chemotherapy versus Cetuximab plus Chemotherapy		140			
36	Finn RS, *et al.* 2020 [64]	NCT03434379	Atezolizumab (PD-L1)	Atezolizumab plus Bevacizumab versus Sorafenib	485	68	No	III	Unresectable hepatocellular carcinoma
37	Gutzmer R, *et al.* 2020 [65]	NCT02908672 (IMspire150)	Atezolizumab (PD-L1)	Atezolizumab plus Vemurafenib plus Cobimetinib versus Vemurafenib plus Cobimetinib	511	209	No	III	Unresectable advanced BRAFV600 mutation-positive melanoma
38	Mittendorf EA, *et al.* 2020 [66]	NCT03197935 (IMpassion031)	Atezolizumab (PD-L1)	Atezolizumab + Chemotherapy versus Chemotherapy	331	88	No	III	Early stage TNBC
39	Ascierto PA, *et al.* 2020 [67]	NCT02388906 (CheckMate 238)	Nivolumab (PD-1)	Nivolumab versus Ipilimumab	905	197	No	III	Resected stage IIIB–C and stage IV Melanoma
40	Herbst RS, *et al.* 2020 [68]	NCT02409342 (IMpower110)	Atezolizumab (PD-L1)	Atezolizumab versus Chemotherapy (platinum-based)	549	63	No	III	PD-L1-selected NSCLC
41	Emens LA, *et al.* 2020 [69]	NCT02924883 (KATE2)	Atezolizumab (PD-L1)	Atezolizumab plus Trastuzumab emtansine versus Trastuzumab emtansine	200	34	Yes	II	HER2-positive advanced breast cancer
42	Huang J, *et al.* 2020 [70]	NCT03099382 (ESCORT)	Camrelizumab (PD-1)	Camrelizumab versus Chemotherapy (Docetaxel or Irinotecan)	448	189	Yes	III	Advanced or metastatic OSCC
43	Powles, *et al.* 2020 [71]	NCT02603432 (JAVELIN Bladder 100)	Avelumab (PD-L1)	Avelumab versus Best Supportive Care (BSC)	689	44	Yes	III	Advanced or metastatic UC
44	André T, *et al.* 2020 [72]	NCT02563002 (KEYNOTE-177)	Pembrolizumab (PD-1)	Pembrolizumab versus Chemotherapy (5-fluorouracil–based therapy with or without bevacizumab or cetuximab)	296	36	No	III	Colorectal cancer
45	Schmid P, *et al.* 2020 [73]	NCT03036488 (KEYNOTE-522)	Pembrolizumab (PD-1)	Pembrolizumab plus Chemotherapy (Paclitaxel plus Carboplatin) versus Placebo plus Chemotherapy (Paclitaxel plus Carboplatin)	1170	229	No	III	Stage II or stage III TNBC
46	Jotte R, *et al.* 2020 [74]	NCT02367794 (IMpower131)	Atezolizumab (PD-L1)	Atezolizumab plus Carboplatin plus Nab-paclitaxel versus Carboplatin plus Nab-paclitaxel	668	38	Yes	III	Advanced squamous NSCLC
47	Zhou C, *et al.* 2020 [75]	NCT03134872 (CameL)	Camrelizumab (PD-1)	Camrelizumab plus Carboplatin plus Pemetrexed versus Carboplatin plus Pemetrexed	412	36	No	III	Nonsquamous NSCLC
48	Zimmer L, *et al.* 2020A [76]	NCT02523313 (IMMUNED)	Nivolumab (PD-1)	Nivolumab plus Ipilimumab versus Nivolumab	162	6	Yes	II	Resected stage IV melanoma
	Zimmer L, *et al.* 2020B [76]			Nivolumab plus Ipilimumab versus Placebo		N/A			
	Zimmer L, *et al.* 2020C [76]			Nivolumab versus Placebo		N/A			
49	Galsky MD, *et al.* 2020A [77]	NCT02807636 (IMvigor130)	Atezolizumab (PD-L1)	Atezolizumab plus Chemotherapy (platinum-based) versus Atezolizumab	1203	75	No	III	Locally advanced or metastatic UC
	Galsky MD, *et al.* 2020B [77]			Atezolizumab plus Chemotherapy versus Chemotherapy		80			
	Galsky MD, *et al.* 2020C [77]			Atezolizumab versus Placebo plus Chemotherapy		41			
50	Powles T, *et al.* 2020A [78]	NCT02516241 (DANUBE)	Durvalumab (PD-L1)	Durvalumab versus Durvalumab plus Tremelimumab	998	73	No	III	Unresectable advanced or metastatic UC
	Powles T, *et al.* 2020B [78]			Durvalumab versus Chemotherapy (gemcitabine plus cisplatin/carboplatin)		34			
51	Rudin CM, *et al.* 2020 [79]	NCT03066778 (KEYNOTE-604)	Pembrolizumab (PD-1)	Pembrolizumab plus EP versus Placebo plus EP	446	43	No	III	Extensive-stage SCLC
52	Shitara K, *et al.* 2020A [80]	NCT02494583 (KEYNOTE-062)	Pembrolizumab (PD-1)	Pembrolizumab versus Pembrolizumab plus Chemotherapy (Cisplatin plus Fluorouracil/Capecitabine)	748	43	No	III	Advanced GC
	Shitara K, *et al.* 2020B [80]			Pembrolizumab versus Chemotherapy		26			
	Shitara K, *et al.* 2020C [80]			Pembrolizumab plus Chemotherapy versus Chemotherapy		37			
53	Ribas A, *et al.* 2020A [81]	NCT02027961	Durvalumab (PD-L1)	Durvalumab plus Dabrafenib plus Trametinib versus Durvalumab plus Trametinib (concurrent)	68	16	Yes	I	Advanced melanoma
	Ribas A, *et al.* 2020B [81]			Durvalumab plus Dabrafenib plus Trametinib versus Durvalumab plus Trametinib (sequential)		20			
	Ribas A, *et al.* 2020C [81]			Durvalumab plus Trametinib (concurrent) versus Durvalumab plus Trametinib (sequential)		18			
54	Winer EP, *et al.* 2021 [82]	NCT02555657 (KEYNOTE-119)	Pembrolizumab (PD-1)	Pembrolizumab versus Single-drug Chemotherapy	601	8	Yes	III	Metastatic TNBC
55	Lee NY, *et al.* 2021 [83]	NCT02952586	Avelumab (PD-L1)	Avelumab plus Chemoradiotherapy versus placebo plus Chemoradiotherapy	692	56	No	III	Locally advanced HNSCC
56	Miles D, *et al.* 2021 [84]	NCT03125902 (IMpassion131)	Atezolizumab (PD-L1)	Atezolizumab plus Paclitaxel versus Placebo plus Paclitaxel	649	207	No	III	Locally advanced/metastatic TNBC
57	Ren Z, *et al.* 2021 [85]	NCT03794440 (ORIENT-32)	Sintilimab (PD-1)	Sintilimab plus Bevacizumab biosimilar (IBI305) versus sorafenib	565	49	No	II-III	Unresectable hepatocellular carcinoma
58	Powles T, *et al.* 2021A [86]	NCT02853305 (KEYNOTE-361)	Pembrolizumab (PD-1)	Pembrolizumab versus Chemotherapy	993	64	No	III	Advanced UC
	Powles T, *et al.* 2021B [86]			Pembrolizumab plus Chemotherapy versus Chemotherapy		107			
	Powles T, *et al.* 2021C [86]			Pembrolizumab versus Pembrolizumab plus Chemotherapy		123			
59	Bajorin DF, *et al.* 2021 [87]	NCT02632409 (CheckMate 274)	Nivolumab (PD-1)	Nivolumab (Adjuvant) versus placebo	699	72	Yes	III	Muscle-invasive UC
60	Brufsky A, *et al.* 2021A [88]	NCT02322814 (COLET)	Atezolizumab (PD-L1)	Cobimetinib plus atezolizumab plus paclitaxel versus Cobimetinib plus paclitaxel	152	32	No	II	Advanced or metastatic TNBC
	Brufsky A, *et al.* 2021B [88]			Cobimetinib plus atezolizumab plus paclitaxel versus Cobimetinib plus atezolizumab plus Nab-paclitaxel		28			
	Brufsky A, *et al.* 2021C [88]			Cobimetinib plus paclitaxel versus Placebo plus paclitaxel		25			
61	Motzer R, *et al.* 2021A [89]	NCT02811861 (CLEAR)	Pembrolizumab (PD-1)	Lenvatinib plus Pembrolizumab versus Sunitinib	1047	143	No	III	Advanced RCC
	Motzer R, *et al.* 2021B [89]			Lenvatinib plus Pembrolizumab versus Lenvatinib plus Everolimus		184			
	Motzer R, *et al.* 2021C [89]			Lenvatinib plus Everolimus versus Sunitinib		135			
62	Bellmunt J, *et al.* 2021 [90]	NCT02450331 (IMvigor010)	Atezolizumab (PD-L1)	Atezolizumab versus Observation	787	101	No	III	Muscle-invasive UC
63	Choueiri TK, *et al.* 2021 [91]	NCT03141177 (CheckMate 9ER)	Nivolumab (PD-1)	Nivolumab plus Cabozantinib versus Sunitinib	640	95	No	III	Advanced RCC
64	Sezer A, *et al.* 2021 [92]	NCT03088540 (EMPOWER-Lung 1)	Cemiplimab (PD-1)	Cemiplimab versus Chemotherapy (platinum-doublet)	697	26	No	III	Advanced NSCLC
65	Paz-Ares L, *et al.* 2021 [93]	NCT03215706 (CheckMate 9LA)	Nivolumab (PD-1)	Nivolumab plus Ipilimumab plus Chemotherapy versus Chemotherapy	707	78	No	III	Stage IV or recurrent NSCLC
66	Baas P, *et al.* 2021 [94]	NCT02899299 (CheckMate 743)	Nivolumab (PD-1)	Nivolumab plus Ipilimumab versus Chemotherapy	584	58	No	III	Unresectable malignant pleural mesothelioma
67	Goldman JW, *et al.* 2021A [95]	NCT03043872 (CASPIAN)	Durvalumab (PD-L1)	Durvalumab plus EP versus EP	797	26	No	III	Extensive-stage SCLC
	Goldman JW, *et al.* 2021B [95]			Durvalumab plus Tremelimumab (CTLA-4) plus EP versus EP		46			
	Goldman JW, *et al.* 2021C [95]			Durvalumab plus Tremelimumab (CTLA-4) plus EP versus Durvalumab plus EP		52			
68	Pujade-Lauraine E, *et al.* 2021A [96]	NCT02580058 (JAVELIN Ovarian 200)	Avelumab (PD-L1)	Avelumab plus PLD (Pegylated Liposomal Doxorubicin) versus PLD	546	61	Yes	III	Platinum-resistant or platinum-refractory OC
	Pujade-Lauraine E, *et al.* 2021B [96]			Avelumab plus PLD versus Avelumab		54			
	Pujade-Lauraine E, *et al.* 2021C [96]			Avelumab versus PLD		25			
69	Kelly RJ, *et al.* 2021 [97]	NCT02743494 (CheckMate 577)	Nivolumab (PD-1)	Nivolumab versus Placebo	792	62	Yes	III	Resected esophageal or GJC
70	Sugawara S, *et al.* 2021 [98]	NCT03117049 (ONO-4538-52/TASUKI-5)	Nivolumab (PD-1)	Nivolumab versus Placebo	548	121	No	III	Stage IIIB/IV or recurrent nonsquamous NSCLC
71	Yang Y, *et al.* 2021 [99]	NCT03707509 (CAPTAIN-1st)	Camrelizumab (PD-1)	Camrelizumab plus Gemcitabine plus Cisplatin versus Gemcitabine plus Cisplatin	263	72	No	III	NC
72	Liu SV, *et al.* 2021 [100]	NCT02763579 (IMpower133)	Atezolizumab (PD-L1)	Atezolizumab plus CP/ET versus Placebo plus CP/ET	394	61	No	I/III	Extensive-stage SCLC
73	Monk BJ, *et al.* 2021A [101]	NCT02718417 (JAVELIN Ovarian 100)	Avelumab (PD-L1)	Avelumab plus Chemotherapy + Avelumab (maintenance) versus Chemotherapy	991	91	No	III	Stage III–IV epithelial OC
	Monk BJ, *et al.* 2021B [101]			Avelumab plus Chemotherapy plus Avelumab (maintenance) versus Chemotherapy plus Avelumab (maintenance)		125			
	Monk BJ, *et al.* 2021C [101]			Chemotherapy plus Avelumab (maintenance) versus Chemotherapy		84			
74	Choueiri TK, *et al.* 2021 [102]	NCT03142334 (KEYNOTE-564)	Pembrolizumab (PD-1)	Pembrolizumab versus Placebo	984	151	No	III	Clear-cell, advanced RCC
75	Moore KN, *et al.* 2021 [103]	(NCT03038100) (IMagyn050/GOG 3015/ENGOT-OV39)	Atezolizumab (PD-L1)	Atezolizumab plus CP plus Bevacizumab versus Placebo plus CP plus Bevacizumab	1285	252	No	III	Stage III or IV OC
76	Gogas H, *et al.* 2021 [104]	NCT03273153 (IMspire170)	Atezolizumab (PD-L1)	Cobimetinib plus Atezolizumab versus Pembrolizumab	436	118	No	III	BRAFV600 wild-type melanoma
77	Owonikoko TK, *et al.* 2021A [105]	NCT02538666 (CheckMate 451)	Nivolumab (PD-1)	Nivolumab plus Ipilimumab versus Nivolumab	830	82	Yes	III	Extensive-disease SCLC
	Owonikoko TK, *et al.* 2021B [105]			Nivolumab plus Ipilimumab versus Placebo		76			
	Owonikoko TK, *et al.* 2021C [105]			Nivolumab versus Placebo		28			
78	Luo H, *et al.* 2021 [106]	NCT03691090 ((ESCORT-1st)	Camrelizumab (PD-1)	Camrelizumab plus Chemotherapy versus Chemotherapy	595	22	No	III	Advanced or metastatic ESCC
79	Colombo N, *et al.* 2021A [107]	NCT03635567 (KEYNOTE-826)	Pembrolizumab (PD-1)	Pembrolizumab plus Chemotherapy plus Bevacizumab versus Chemotherapy plus Bevacizumab	389	65	No	III	Persistent, recurrent, or metastatic cervical cancer
	Colombo N, *et al.* 2021B [107]			Pembrolizumab plus Chemotherapy versus Chemotherapy	227	17			
80	Fennell DA, *et al.* 2021 [108]	NCT03063450 (CONFIRM)	Nivolumab (PD-1)	Nivolumab versus Placebo	332	1	Yes	III	Malignant mesothelioma
81	Pusztai L, *et al.* 2021 [109]	(NCT01042379) (I-SPY2)	Durvalumab (PD-L1)	Durvalumab plus Olaparib plus Paclitaxel (DOP) versus Paclitaxel	372	63	No	II	HER2-negative stage II/III breast cancer
82	Zhu X, *et al.* 2021 [110]	NCT02704156	Pembrolizumab (PD-1)	SBRT plus Pembrolizumab plus Trametinib versus SBRT plus Gemcitabine	170	22	Yes	II	Locally recurrent pancreatic cancer after surgical resection
83	Sun JM, *et al.* 2021 [111]	NCT03189719 (KEYNOTE-590)	Pembrolizumab (PD-1)	Pembrolizumab plus Chemotherapy versus Placebo plus Chemotherapy	740	47	No	III	Advanced esophageal cancer
84	Mai HQ, *et al.* 2021 [112]	NCT03581786	Toripalimab (PD-1)	Toripalimab plus GP versus Placebo plus GP	289	71	No	III	Advanced NC
85	Felip E, *et al.* 2021 [113]	NCT02486718 (IMpower010)	Atezolizumab (PD-L1)	Atezolizumab versus BSC	990	102	Yes	III	Resected stage IB–IIIA NSCLC
86	Larkin J, *et al.* 2019A [114]	NCT01844505 (CheckMate 067)	Nivolumab (PD-1)	Nivolumab plus Ipilimumab versus Nivolumab	937	167	No	III	Advanced melanoma
	Larkin J, *et al.* 2019B [114]			Nivolumab plus Ipilimumab versus Ipilimumab		162			
	Larkin J, *et al.* 2019C [114]			Nivolumab versus Ipilimumab		143			
	Wolchok JD, *et al.* 2017A [115]			Nivolumab plus Ipilimumab versus Nivolumab		165			
	Wolchok JD, *et al.* 2017B [115]			Nivolumab plus Ipilimumab versus Ipilimumab		161			
	Wolchok JD, *et al.* 2017C [115]			Nivolumab versus Ipilimumab		140			
	Hodi FS, *et al.* 2018A [116]			Nivolumab plus Ipilimumab versus Nivolumab		167			
	Hodi FS, *et al.* 2018B [116]			Nivolumab plus Ipilimumab versus Ipilimumab		162			
	Hodi FS, *et al.* 2018C [116]			Nivolumab versus Ipilimumab		143			
	Larkin J, *et al.* 2015A [117]			Nivolumab versus Nivolumab plus Ipilimumab		207			
	Larkin J, *et al.* 2015B [117]			Nivolumab versus Ipilimumab		183			
	Larkin J, *et al.* 2015C [117]			Nivolumab plus Ipilimumab versus Ipilimumab		228			

RCT = randomized controlled trial, N/A = not available, NSCLC=non-small-cell lung cancer, SCLC = small-cell lung cancer, UC = urothelial carcinoma, HNSCC = head and neck squamous-cell carcinoma, TNBC = triple-negative breast cancer, NC = nasopharyngeal carcinoma, GJC = gastroesophageal junction cancer, GC = gastric cancer, ESCC = esophageal squamous cell carcinoma, OC= ovarian cancer, and RCC = renal cell carcinoma.

## Data Availability

The data used to support the findings of this study are included within the article.

## Abbreviations

PRISMA: Preferred Reporting Items for Systematic Reviews and Meta-Analyses
PICOS: Participants, interventions, comparisons, outcomes, and study design
RCT: Randomized controlled trial
N/A: Not available
PD-1: Programmed cell death-1
PD-L1: Programmed cell death ligand 1
HR: Hazard ratios
OR: Odds ratio
RD: Risk difference
CI: Confidence interval
RE: Random effect
NSCLC: Non-small-cell lung cancer
SCLC: Small-cell lung cancer
NC: Nasopharyngeal carcinoma
OC: Ovarian cancer
TNBC: Triple-negative breast cancer
HNSCC: Head and neck squamous cell carcinoma
UC: Urothelial carcinoma
GC/GJC: Gastric or gastro-oesophageal junction cancer
RCC: Renal cell carcinoma
ESCC: Esophageal squamous cell carcinoma.

## Supplementary Materials

Supplementary Materials S Figure 1: a summary table of review authors' judgements for each risk of bias item for each study. S Figure 2: funnel plots of comparison in Group A (PD-1 or PD-L1 versus Chemotherapy). A: the OR of rash for all-grade checked using the fixed effect (FE) model: Subgroup analyses were carried out according to the types of immune checkpoint inhibitors (PD-1 or PD-L1). B: the OR of rash for all-grade checked using the fixed effect (FE) model: subgroup analyses were carried out according to the treatment lines (first or second line). C: the OR of rash for all grades checked using the fixed effect (FE) model: Subgroup analyses were carried out based on drug name, tumor type, and immune checkpoint type. D: the OR of rash for all grades checked using the fixed effect (FE) model: subgroup analyses were carried out based on drug name, tumor type, immune checkpoint type, and I^2^ value. S Figure 3: funnel plots of comparison in combination regimens. A: the odds ratio of rash for all grades checked using the fixed effect (FE) model in Group B (PD-1 or PD-L1 plus Chemotherapy versus Chemotherapy): subgroup analyses were carried out according to the types of immune checkpoint inhibitors (PD-1 or PD-L1). B: the odds ratio of rash for all-grade checked using the fixed effect (FE) model in Group B (PD-1 or PD-L1 plus Chemotherapy versus Chemotherapy): subgroup analyses were carried out according to the treatment lines (first or second line). C: the odds ratio of rash for all grades checked using the fixed effect (FE) model in Group B (PD-1 or PD-L1 plus Chemotherapy versus Chemotherapy): subgroup analyses were carried out based on tumor type. D: the odds ratio of rash for all grades checked using the fixed effect (FE) model in Group B (PD-1 or PD-L1 plus Chemotherapy versus Chemotherapy): subgroup analyses were carried out based on tumor type and immune checkpoint type. E: the odds ratio of rash for all grades checked using the fixed effect (FE) model in Group C (Camrelizumab plus Chemotherapy versus Chemotherapy). F: the odds ratio of rash for all-grade checked using the fixed effect (FE) model in Group D (PD-1 or PD-L1 plus Chemotherapy plus Bevacizumab versus Chemotherapy plus Bevacizumab). S Figure 4: funnel plots of different comparisons. A: the OR of rash for all-grade checked using the fixed effect (FE) model in Group E (PD-1 or PD-L1 versus Placebo): subgroup analyses were carried out according to the types of immune checkpoint inhibitors (PD-1 or PD-L1). B: the OR of rash for all grades checked using the fixed effect (FE) model in Group E (PD-1 or PD-L1 versus Placebo): subgroup analyses were carried out according to the treatment lines (first or second line). C: the OR of rash for all grades checked using the fixed effect (FE) model in Group E (PD-1 or PD-L1 versus Placebo): subgroup analyses were carried out based on tumor type. D: the OR of rash for all grades checked using the fixed effect (FE) model in Group E (PD-1 or PD-L1 versus Placebo): subgroup analyses were carried out based on tumor type, and I^2^ value. E: the OR of rash for all grades checked using the fixed effect (FE) model in Group F (PD-1 or PD-L1 plus Chemotherapy versus PD-1 or PD-L1): subgroup analyses were carried out according to the types of immune checkpoint inhibitors (PD-1 or PD-L1). F: the OR of rash for all grades checked using the fixed effect (FE) model in Group F (PD-1 or PD-L1 plus Chemotherapy versus PD-1 or PD-L1): subgroup analyses were carried out according to the treatment lines (first or second line). S Figure 5: funnel plots of comparison groups (Groups G-M). A: the OR of rash for all grades checked using the fixed effect (FE) model in Group G (PD-1 or PD-L1 plus CTLA-4 versus PD-1 or PD-L1): subgroup analyses were carried out according to the types of immune checkpoint inhibitors (PD-1 or PD-L1). B: the OR of rash for all grades checked using the fixed effect (FE) model in Group G (PD-1 OR PD-L1 plus CTLA-4 versus PD-1 or PD-L1): subgroup analyses were carried out based on tumor type. C: the OR of rash for all grades checked using the fixed effect (FE) model in Group G (PD-1 or PD-L1 plus CTLA-4 versus PD-1 or PD-L1): subgroup analyses were carried out according to the treatment lines (first or second line). D: the OR of rash for all grades checked using the fixed effect (FE) model in Group H (PD-1 or PD-L1 versus CTLA-4). E: the OR of rash for all grades checked using the fixed effect (FE) model in Group H (PD-1 or PD-L1 versus CTLA-4): subgroup analyses were carried out according to the treatment lines (first or second line). F: the OR of rash for all grades checked using the fixed effect (FE) model in Group I (PD-1 or PD-L1 plus CTLA-4 versus Chemotherapy): subgroup analyses were carried out based on tumor type. G: the OR of rash for all grades checked using the fixed effect (FE) model in Group J (PD-1 or PD-L1 plus CTLA-4 plus Chemotherapy versus Chemotherapy): subgroup analyses were carried out based on treatment regimens. H: the OR of rash for all grades checked using the fixed effect (FE) model in Group K (PD-1 or PD-L1 plus Bevacizumab versus Sorafenib): subgroup analyses were carried out according to the types of immune checkpoint inhibitors (PD-1 or PD-L1). I: the OR of rash for all grades checked using the fixed effect (FE) model in Group L (PD-1 or PD-L1 plus CTLA-4 versus CTLA-4). J: the OR of rash for all grades checked using the fixed effect (FE) model in Group M (PD-1 or PD-L1 versus Methotrexate/docetaxel/cetuximab). S Figure 6: funnel plots of comparison groups for grades 3–5. A: the OR of rash for grades 3–5 checked using the fixed effect (FE) model in Group A (PD-1 or PD-L1 versus Chemotherapy): subgroup analyses were carried out based on tumor types. B: the OR of rash for grades 3–5 checked using the fixed effect (FE) model in Group B (PD-1 or PD-L1 plus Chemotherapy versus Chemotherapy): subgroup analyses were carried out based on tumor types. C: The OR of rash for grades 3–5 checked using the fixed effect (FE) model in Group E (PD-1 or PD-L1 versus Placebo): subgroup analyses were carried out based on tumor types. D: the OR of rash for grades 3–5 checked using the fixed effect (FE) model in Group G (PD-1 or PD-L1 plus CTLA-4 versus PD-1 or PD-L1): subgroup analyses were carried out based on tumor types. E: the OR of rash for grades 3–5 checked using the fixed effect (FE) model in Group F (PD-1 or PD-L1 plus Chemotherapy versus PD-1 or PD-L1): subgroup analyses were carried out based on the types of immune checkpoint inhibitors (PD-1 or PD-L1). F: the OR of rash for grades 3–5 checked using the fixed effect (FE) model in Group H (PD-1 or PD-L1 versus CTLA-4). G: the OR of rash for grades 3–5 checked using the fixed effect (FE) model in Group M (PD-1 or PD-L1 versus Methotrexate/docetaxel/cetuximab). H: the OR of rash for grades 3–5 checked using the fixed effect (FE) model in Group K (PD-1 or PD-L1 plus Bevacizumab versus Sorafenib): subgroup analyses were carried out based on the name of immune checkpoint inhibitors. I: the OR of rash for grades 3–5 checked using the fixed effect (FE) model in Group J (PD-1 or PD-L1 plus CTLA-4 plus Chemotherapy versus Chemotherapy): subgroup analyses were carried out based on treatment regimens.
