# Supplementary material for: Risk of Rash in PD-1 or PD-L1-Related Cancer Clinical Trials: A Systematic Review and Meta-Analysis
Source: J Oncol. 2022 Jul 18;2022:4976032. doi: 10.1155/2022/4976032 (PMC9313907; doi:10.1155/2022/4976032)

**S Figure 1：**A summary table of review authors' judgements for each risk of bias item for each study.


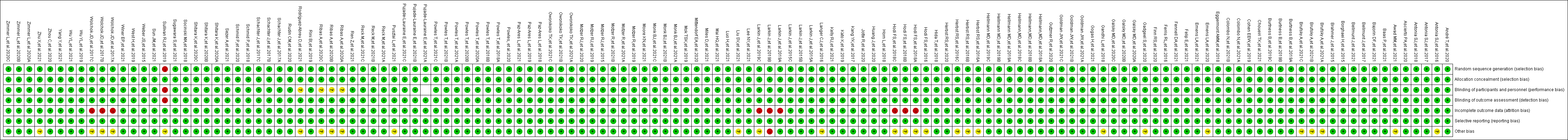


**S Figure 2: Funnel plots of comparison in Group A (PD-1/PD-L1 VS Chemotherapy)**

A: The odds ratio of rash for all-grade calculated by the fixed effect (FE) model: Subgroup analyses was performed according to the types of immune checkpoint inhibitors (PD-1 or PD-L1).

B: The odds ratio of rash for all-grade calculated by the fixed effect (FE) model: Subgroup analyses was performed according to the treatment lines (First or Second line).

C: The odds ratio of rash for all-grade calculated by the fixed effect (FE) model: Subgroup analyses was performed based on drug name, tumor type, and immune checkpoint type.

D: The odds ratio of rash for all-grade calculated by the fixed effect (FE) model: Subgroup analyses was performed based on drug name, tumor type, immune checkpoint type and I^2^ value.


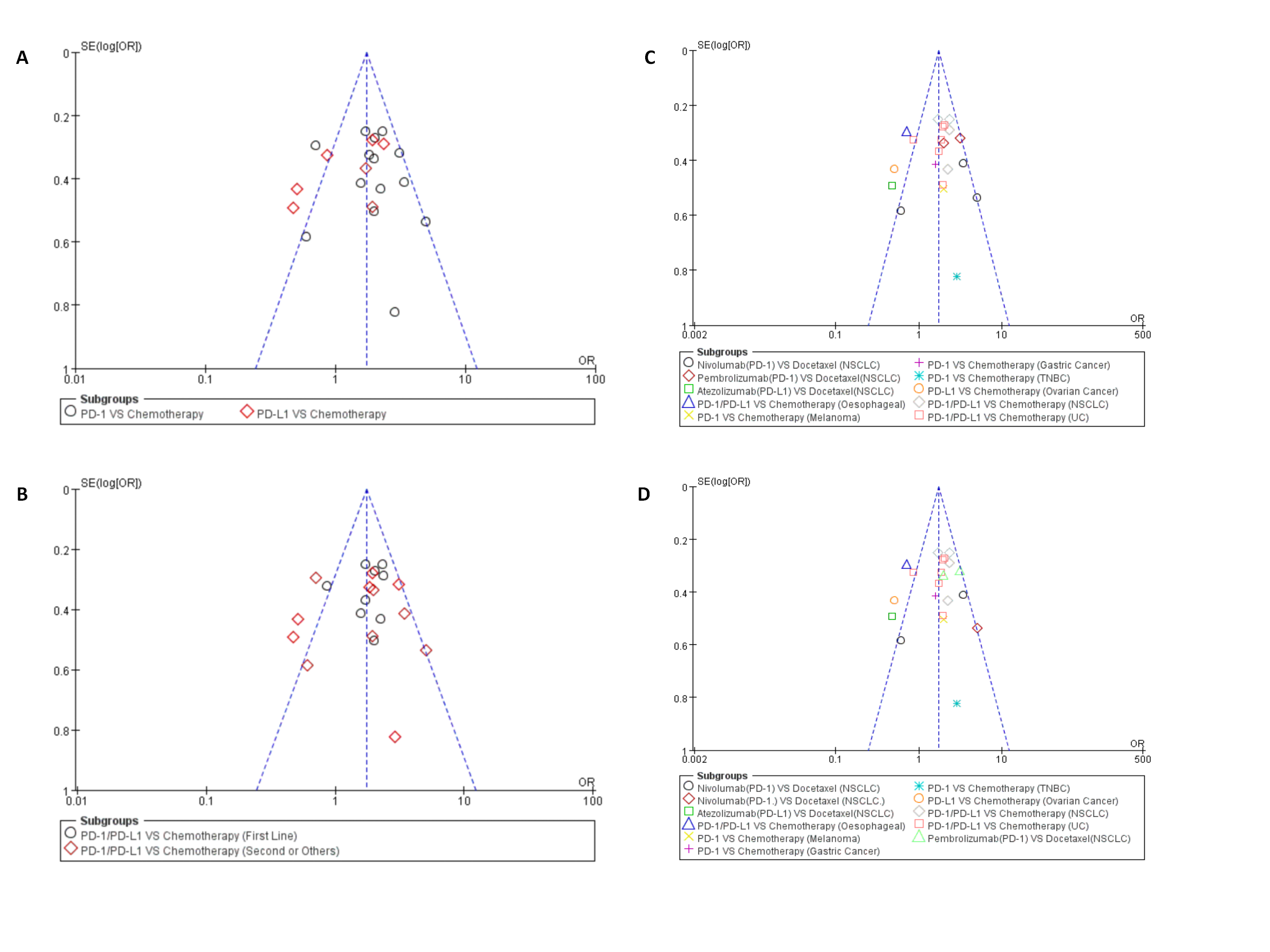


**S Figure 3: Funnel plots of comparison in combination regimens.**

A: The odds ratio of rash for all-grade calculated by the fixed effect (FE) model in Group B (PD-1/PD-L1 + Chemotherapy VS. Chemotherapy): Subgroup analyses were performed according to the types of immune checkpoint inhibitors (PD-1 or PD-L1).

B: The odds ratio of rash for all-grade calculated by the fixed effect (FE) model in Group B (PD-1/PD-L1 + Chemotherapy VS. Chemotherapy): Subgroup analyses were performed according to the treatment lines (First or Second line).

C: The odds ratio of rash for all-grade calculated by the fixed effect (FE) model in Group B (PD-1/PD-L1 + Chemotherapy VS. Chemotherapy): Subgroup analyses were performed based on tumor type.

D: The odds ratio of rash for all-grade calculated by the fixed effect (FE) model in Group B (PD-1/PD-L1 + Chemotherapy VS. Chemotherapy): Subgroup analyses were performed based on tumor type, and immune checkpoint type.

E: The odds ratio of rash for all-grade calculated by the fixed effect (FE) model in Group C (Camrelizumab + Chemotherapy VS. Chemotherapy).

F: The odds ratio of rash for all-grade calculated by the fixed effect (FE) model in Group D (PD-1/PD-L1 + Chemotherapy + Bevacizumab VS. Chemotherapy + Bevacizumab).


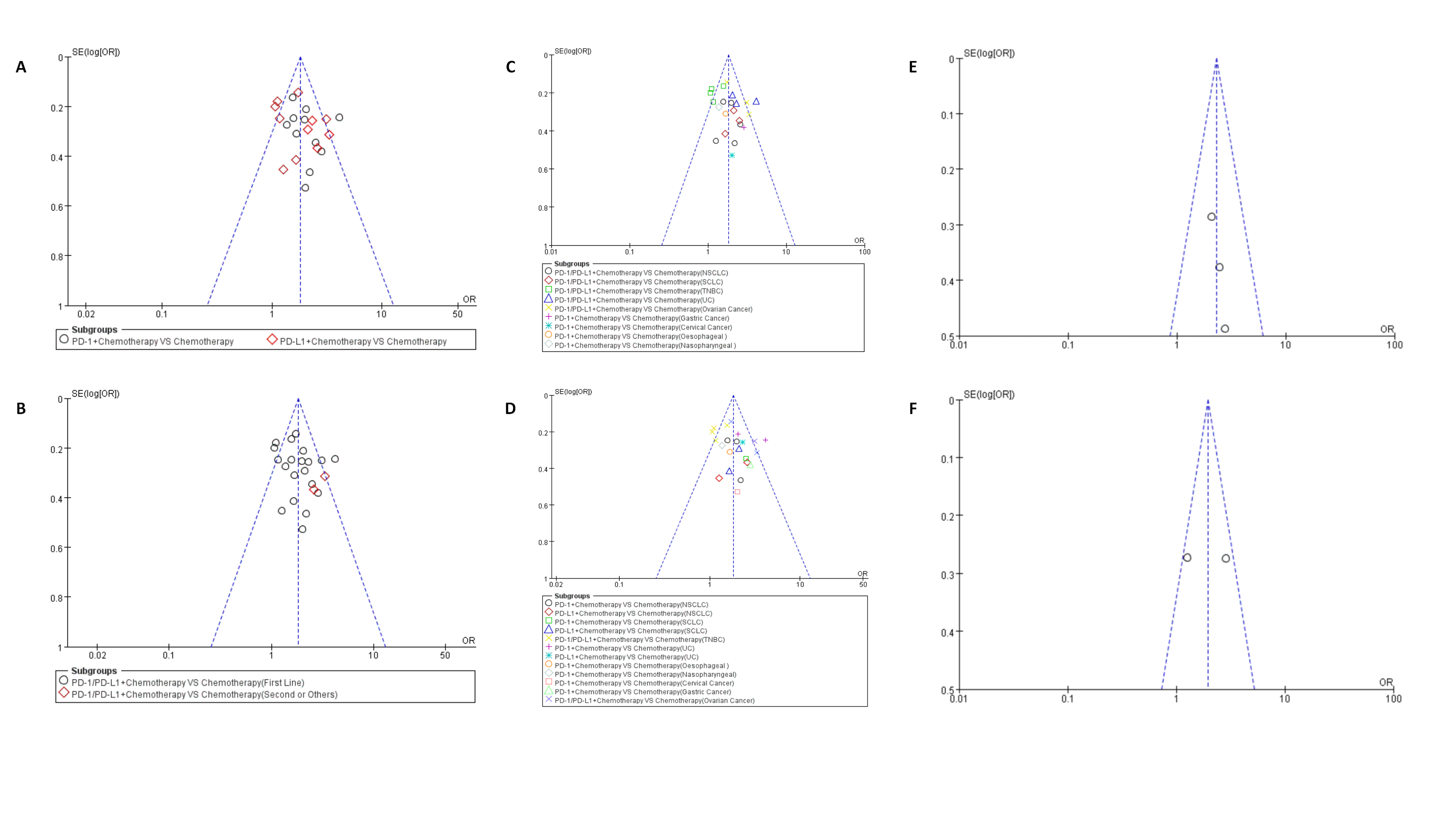


**S Figure 4: Funnel plots of different comparisons.**

A: The odds ratio of rash for all-grade calculated by the fixed effect (FE) model in Group E (PD-1/PD-L1 VS. Placebo): Subgroup analyses were performed according to the types of immune checkpoint inhibitors (PD-1 or PD-L1).

B: The odds ratio of rash for all-grade calculated by the fixed effect (FE) model in Group E (PD-1/PD-L1 VS. Placebo): Subgroup analyses were performed according to the treatment lines (First or Second line).

C: The odds ratio of rash for all-grade calculated by the fixed effect (FE) model in Group E (PD-1/PD-L1 VS. Placebo): Subgroup analyses were performed based on tumor type.

D: The odds ratio of rash for all-grade calculated by the fixed effect (FE) model in Group E (PD-1/PD-L1 VS. Placebo): Subgroup analyses were performed based on tumor type, and I^2^ value.

E: The odds ratio of rash for all-grade calculated by the fixed effect (FE) model in Group F (PD-1/PD-L1+Chemotherapy VS PD-1/PD-L1): Subgroup analyses were performed according to the types of immune checkpoint inhibitors (PD-1 or PD-L1).

F: The odds ratio of rash for all-grade calculated by the fixed effect (FE) model in Group F (PD-1/PD-L1+Chemotherapy VS PD-1/PD-L1): Subgroup analyses were performed according to the treatment lines (First or Second line).


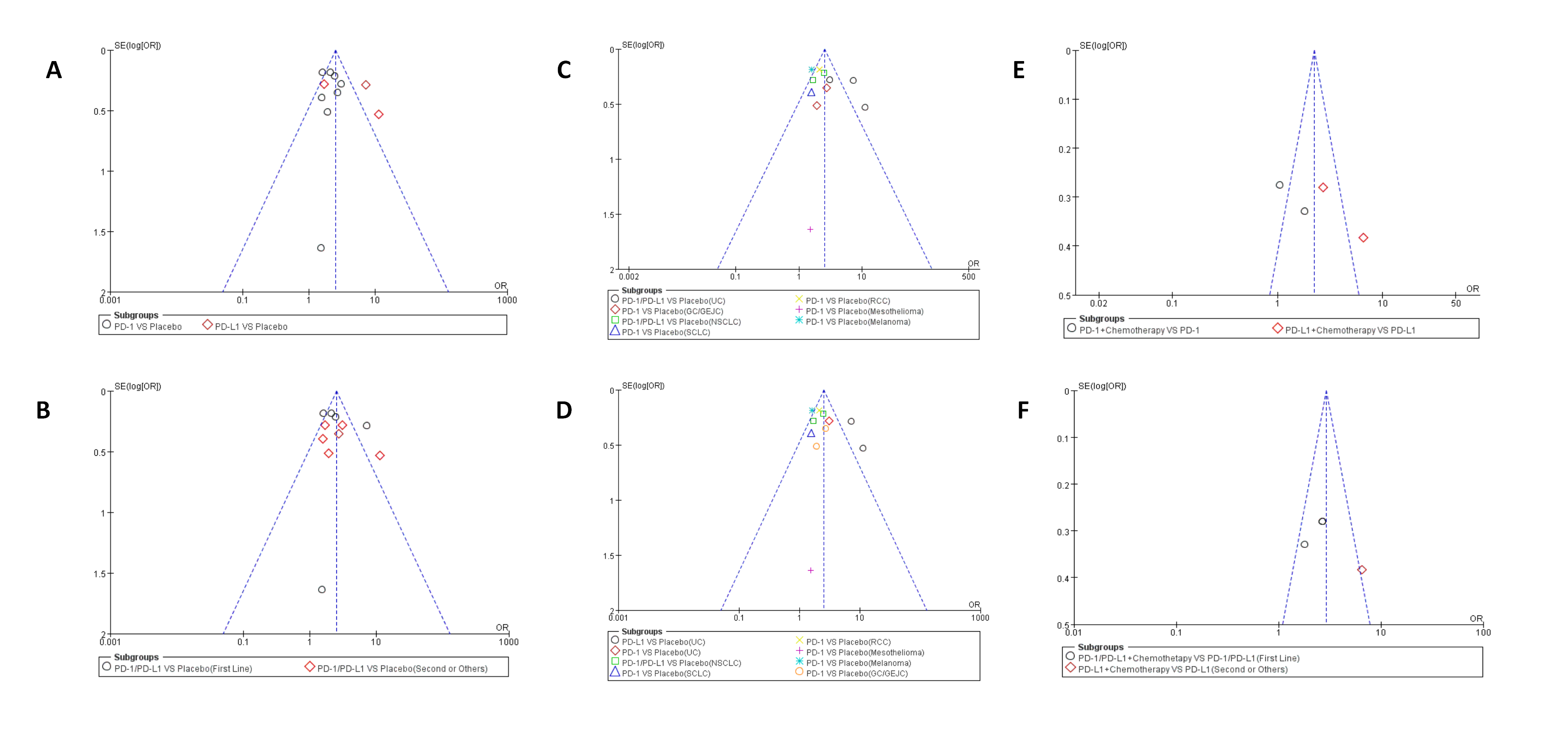


**S Figure 5: Funnel plots of comparison groups (Group G-M).**

A: The odds ratio of rash for all-grade calculated by the fixed effect (FE) model in Group G (PD-1/PD-L1 + CTLA-4 VS. PD-1/PD-L1): Subgroup analyses were performed according to the types of immune checkpoint inhibitors (PD-1 or PD-L1).

B: The odds ratio of rash for all-grade calculated by the fixed effect (FE) model in Group G (PD-1/PD-L1 + CTLA-4 VS. PD-1/PD-L1): Subgroup analyses were performed based on tumor type.

C: The odds ratio of rash for all-grade calculated by the fixed effect (FE) model in Group G (PD-1/PD-L1 + CTLA-4 VS. PD-1/PD-L1): Subgroup analyses were performed according to the treatment lines (First or Second line).

D: The odds ratio of rash for all-grade calculated by the fixed effect (FE) model in Group H (PD-1/PD-L1 VS. CTLA-4).

E: The odds ratio of rash for all-grade calculated by the fixed effect (FE) model in Group H (PD-1/PD-L1 VS. CTLA-4): Subgroup analyses were performed according to the treatment lines (First or Second line).

F: The odds ratio of rash for all-grade calculated by the fixed effect (FE) model in Group I (PD-1/PD-L1 + CTLA-4 VS. Chemotherapy): Subgroup analyses were performed based on tumor type.

G: The odds ratio of rash for all-grade calculated by the fixed effect (FE) model in Group J (PD-1/PD-L1 + CTLA-4 + Chemotherapy VS. Chemotherapy): Subgroup analyses were performed based on treatment regimens.

H: The odds ratio of rash for all-grade calculated by the fixed effect (FE) model in Group K (PD-1/PD-L1 + Bevacizumab VS. Sorafenib): Subgroup analyses were performed according to the types of immune checkpoint inhibitors (PD-1 or PD-L1).

I: The odds ratio of rash for all-grade calculated by the fixed effect (FE) model in Group L (PD-1/PD-L1+CTLA-4 VS CTLA-4).

J: The odds ratio of rash for all-grade calculated by the fixed effect (FE) model in Group M (PD-1/ PD-L1 VS. Methotrexate/docetaxel/cetuximab).


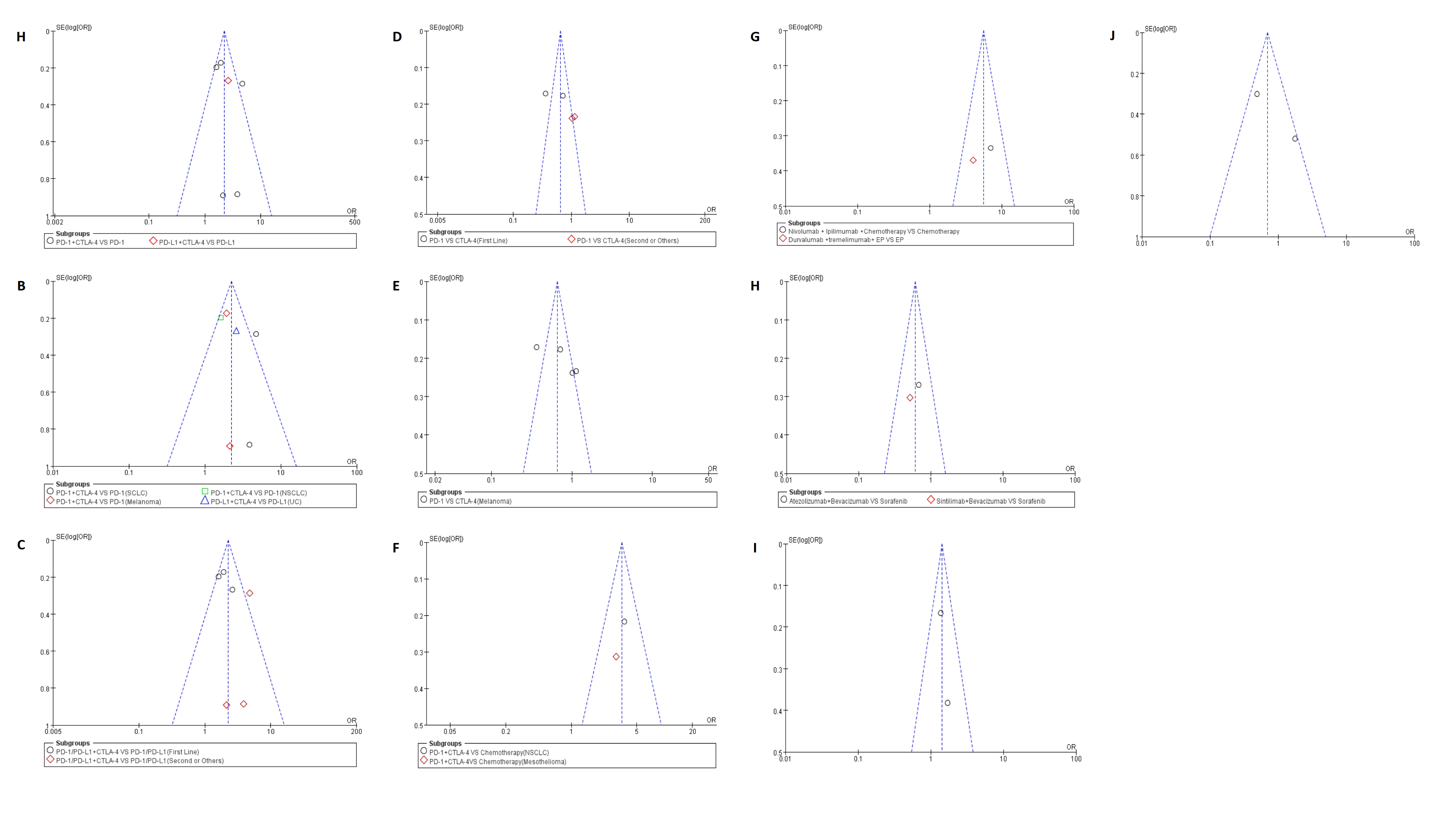


**S Figure 6: Funnel plots of comparison groups for grade 3-5.**

A: The odds ratio of rash for grade 3-5 calculated by the fixed effect (FE) model in Group A (PD-1/PD-L1 VS. Chemotherapy): Subgroup analyses were performed based on tumor types.

B: The odds ratio of rash for grade 3-5 calculated by the fixed effect (FE) model in Group B (PD-1/PD-L1+ Chemotherapy VS. Chemotherapy): Subgroup analyses were performed based on tumor types.

C: The odds ratio of rash for grade 3-5 calculated by the fixed effect (FE) model in Group E (PD-1/PD-L1 VS. Placebo): Subgroup analyses were performed based on tumor types.

D: The odds ratio of rash for grade 3-5 calculated by the fixed effect (FE) model in Group G (PD-1/PD-L1 + CTLA-4 VS. PD-1/PD-L1): Subgroup analyses were performed based on tumor types.

E: The odds ratio of rash for grade 3-5 calculated by the fixed effect (FE) model in Group F (PD-1/PD-L1+Chemotherapy VS PD-1/PD-L1): Subgroup analyses were performed based on the types of immune checkpoint inhibitors (PD-1 or PD-L1).

F: The odds ratio of rash for grade 3-5 calculated by the fixed effect (FE) model in Group H (PD-1/PD-L1 VS. CTLA-4).

G: The odds ratio of rash for grade 3-5 calculated by the fixed effect (FE) model in Group M (PD-1/PD-L1 VS. Methotrexate/docetaxel/cetuximab).

H: The odds ratio of rash for grade 3-5 calculated by the fixed effect (FE) model in Group K (PD-1/PD-L1 + Bevacizumab VS. Sorafenib): Subgroup analyses were performed based on the name of immune checkpoint inhibitors.

I: The odds ratio of rash for grade 3-5 calculated by the fixed effect (FE) model in Group J (PD-1/PD-L1 + CTLA-4 + Chemotherapy VS. Chemotherapy): Subgroup analyses were performed based on treatment regimens.


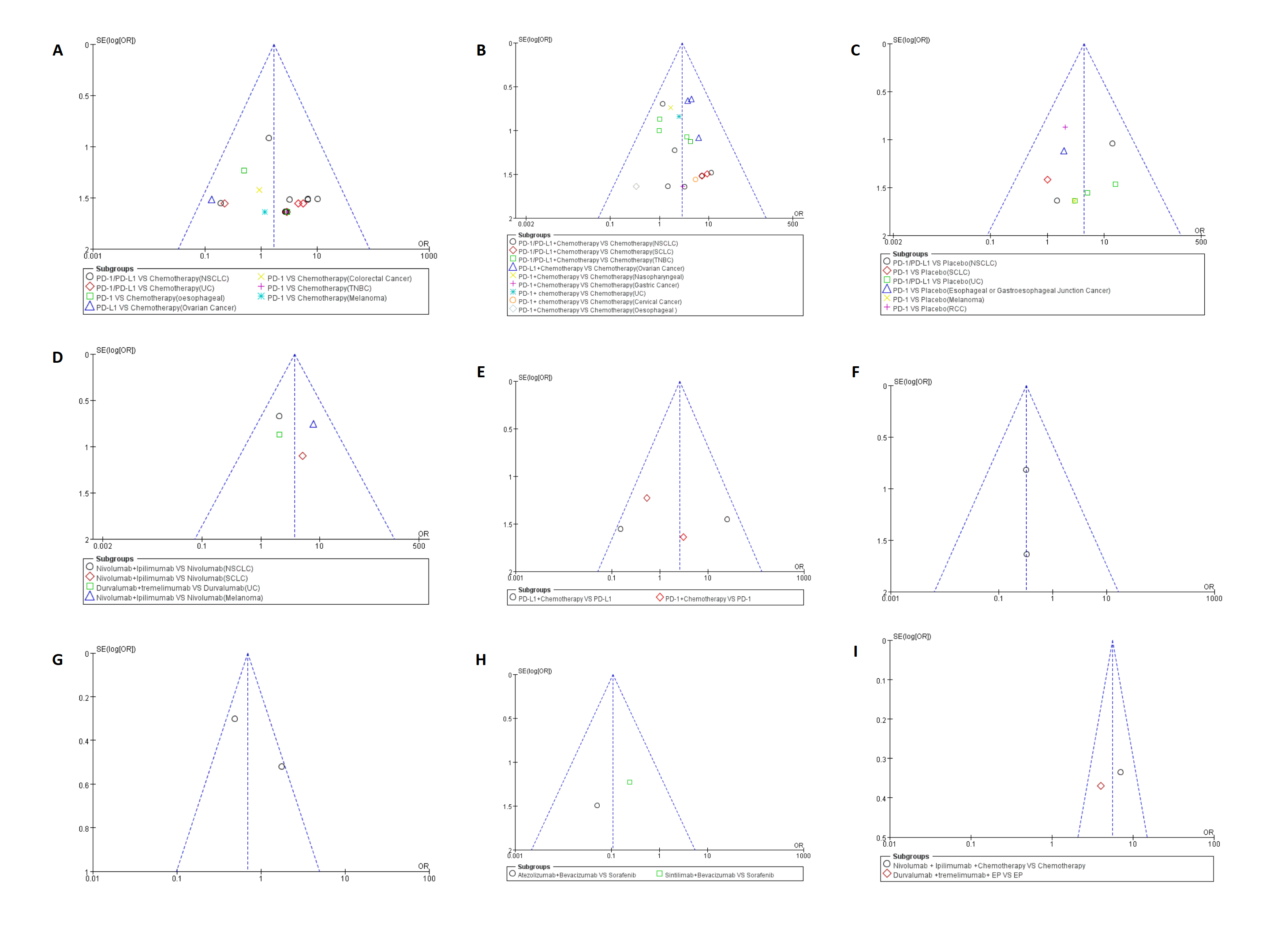

Supplement: Supplementary Materials — S Figure 1: a summary table of review authors' judgements for each risk of bias item for each study. S Figure 2: funnel plots of comparison in Group A (PD-1 or PD-L1 versus Chemotherapy). A: the OR of rash for all-grade checked using the fixed effect (FE) model: Subgroup analyses were carried out according to the types of immune checkpoint inhibitors (PD-1 or PD-L1). B: the OR of rash for all-grade checked using the fixed effect (FE) model: subgroup analyses were carried out according to the treatment lines (first or second line). C: the OR of rash for all grades checked using the fixed effect (FE) model: Subgroup analyses were carried out based on drug name, tumor type, and immune checkpoint type. D: the OR of rash for all grades checked using the fixed effect (FE) model: subgroup analyses were carried out based on drug name, tumor type, immune checkpoint type, and I2 value. S Figure 3: funnel plots of comparison in combination regimens. A: the odds ratio of rash for all grades checked using the fixed effect (FE) model in Group B (PD-1 or PD-L1 plus Chemotherapy versus Chemotherapy): subgroup analyses were carried out according to the types of immune checkpoint inhibitors (PD-1 or PD-L1). B: the odds ratio of rash for all-grade checked using the fixed effect (FE) model in Group B (PD-1 or PD-L1 plus Chemotherapy versus Chemotherapy): subgroup analyses were carried out according to the treatment lines (first or second line). C: the odds ratio of rash for all grades checked using the fixed effect (FE) model in Group B (PD-1 or PD-L1 plus Chemotherapy versus Chemotherapy): subgroup analyses were carried out based on tumor type. D: the odds ratio of rash for all grades checked using the fixed effect (FE) model in Group B (PD-1 or PD-L1 plus Chemotherapy versus Chemotherapy): subgroup analyses were carried out based on tumor type and immune checkpoint type. E: the odds ratio of rash for all grades checked using the fixed effect (FE) model in Group C (Camrelizumab plus C [file 4976032.f1.doc]
